# Job strain and effort–reward imbalance as risk factors for type 2 diabetes mellitus: A systematic review and meta-analysis of prospective studies

**DOI:** 10.5271/sjweh.3987

**Published:** 2021-12-30

**Authors:** Ana Paula B Pena-Gralle, Denis Talbot, Caroline S Duchaine, Mathilde Lavigne-Robichaud, Xavier Trudel, Karine Aubé, Matthias Gralle, Mahée Gilbert-Ouimet, Alain Milot, Chantal Brisson

**Affiliations:** 1 CHU de Québec Research Center, Population Health and Optimal Health Practices Unit, Saint-Sacrément Hospital, Québec, QC, Canada; 2 Faculty of Medicine, Laval University, Ferdinand Vandry Pavillon, Québec, QC, Canada; 3 Department of Medical Biochemistry Leopoldo de Meis, Federal University Rio de Janeiro, Rio de Janeiro, Brazil

**Keywords:** adult-onset diabetes, cohort study, demand–control, ERI, ROBINS-I, work stress

## Abstract

**Objectives:**

This systematic review and meta-analysis aimed to synthesize the available data on prospective associations between work-related stressors and the risk of type 2 diabetes mellitus (T2DM) among adult workers, according to the demand–control–support (DCS) and the effort–reward imbalance (ERI) models.

**Method:**

We searched for prospective studies in PubMed, EMBASE, Web of Science, Scopus, CINHAL and PsychInfo. After screening and extraction, quality of evidence was assessed using the ROBINS-I tool adapted for observational studies. The effect estimates extracted for each cohort were synthesized using random effect models.

**Results:**

We included 18 studies (reporting data on 25 cohorts) in meta-analyses for job strain, job demands, job control, social support at work and ERI. Workers exposed to job strain had a higher risk of developing T2DM when compared to unexposed workers [pooled rate ratio (RR) 1.16, 95% confidence interval (CI) 1.07–1.26]. This association was robust in several supplementary analyses. For exposed women relative to unexposed women, the RR was 1.35 (95% CI 1.12–1.64). The RR of workers exposed to ERI was 1.24 (95% CI 1.08–1.42) compared to unexposed workers.

**Conclusions:**

This is the first meta-analysis to find an effect of ERI on the onset of T2DM incidence. It also confirms that job strain increases the incidence of T2DM, especially among women.

Type 2 diabetes mellitus (T2DM) is a rapidly growing health problem worldwide. The World Health Organization estimates that from 1980 to 2014, worldwide T2DM prevalence among adults rose from 4.7% to 8.5% (1).

Despite an overall increase in life expectancy over the past decades, disability-adjusted life expectancy has not kept up with this gain. Among the elderly, for example, death is often preceded by years of chronic disease (2). In this regard, T2DM currently occupies fourth place among the conditions that most strongly reduce disability-adjusted life years (2).

With the aim of reducing worldwide mortality from chronic diseases by 25% by 2025, the World Health Organization published the 25×25 Global Action Plan (3) which proposes to prevent the increase in the prevalence of T2DM through changes in dietary patterns and physical activity. However, in addition to other major guidelines (4, 5), that plan does not discuss the importance of factors related to the work environment. With the rapid aging of the population, countries in Europe and North America are putting in place incentives for later retirement (6, 7). The population is therefore exposed for a longer period to the work environment, including work-related stressors, and it becomes even more important to consider these as risk factors for chronic diseases.

Work-related stressors are most frequently measured by the demand–control–support (DCS) and effort–reward imbalance (ERI) models. In the DCS model, the combination of high psychosocial demands and low job control, defined as job strain, is the most harmful to health (8). The ERI model assumes that effort at work is spent as part of a contract based on the norm of social reciprocity, where rewards are provided in terms of money, esteem, and career opportunities including job security. It proposes that risks to health arise from a perceived breach of this contract. The perception of this imbalance is affected by personal coping characteristics (overcommitment) (9).

A body of evidence built in recent decades from longitudinal studies in large cohorts of workers has found that work-related stressors, defined according to either of these models, are associated with a moderately higher risk of cardiovascular disease and stroke (10). However, the association between work-related stressors and T2DM remains uncertain: while some studies have found a positive association (11), others have not (12, 13).

The two previous meta-analyses on prospective studies measured work-related stressors only according to the DCS model (14, 15). The most recent meta-analysis differs from the previous one with its inclusion of only one additional study (16). Including this extra study led to observing a significant effect of job strain on T2DM incidence [relative risk 1.16, 95% confidence interval (CI) 1.03–1.31], while the previous meta-analysis had not seen a significant effect (relative risk 1.12, 95% CI 0.95–1.32). This instability casts doubt on the robustness of the findings and warrants further investigation. It is noteworthy that both these meta-analyses are strongly driven by a single aggregated cohort study (11), further decreasing the robustness of the findings.

Here, we have evaluated the risk of bias separately for each cohort using the Risk Of Bias In Non-randomized Studies of Interventions (ROBINS-I) tool (17) adapted for occupational studies (18). The separate evaluation avoids giving any single published study too much weight and avoids counting the same participants more than once when they appear in several published studies.

Furthermore, we included original studies published very recently that have not yet been incorporated into any previous reviews (13, 19, 20). Specifically, enough studies have now been published using the ERI model that we are able to present the first meta-analysis using this model.

## Methods

This review follows the Preferred Reporting Items for Systematic Reviews and Meta-analyses (PRISMA) (21) and the Meta-analysis of Observational Studies in Epidemiology (MOOSE) reporting guidelines (22).

### Inclusion and exclusion criteria

*Eligibility criteria*. Studies were eligible if they were (i) published after 1979 [the date of the first publication using the oldest validated theoretical model considered in this systematic review (DCS)] (23); (ii) measured the exposure using at least one of the two work-related stressor models considered, and (iii) measured the incidence of T2DM.

### Population

The target population of this systematic review included all adult male and female workers. To avoid reverse causality bias, studies involving only sick participants were not considered. Furthermore, studies on pregnant women were excluded because of pregnancy-related traits that may confound the association between work-related stressors and T2DM.

### Exposure

All dimensions of the DCS or ERI models were considered: psychological work demands, job control, social support (from colleagues and/or supervisors) as well as efforts, rewards at work and overcommitment. Combinations of these dimensions were also considered: job strain (high psychological demands combined with low control), iso-strain (job strain combined with low social support) and ERI (ratio between efforts and rewards).

### Comparator

The comparison group had to be from the same study population and a group of workers exposed to the lowest category of the work-related stressors mentioned above.

### Outcomes

The types of incident T2DM considered included clinical measurements [blood glucose, blood insulin, glycated hemoglobin or Homeostatic Model Assessment of Insulin Resistance (HOMA-IR)]; physician-certified or from administrative data (physician services or medication); and self-reported.

### Study design

To avoid the possibility of introducing recall bias and reverse causality, only original studies with prospective design were considered, specifically cohort and nested case–control studies. There was no restriction on the minimum follow-up time.

### Data sources and search strategy

The first author identified articles on 14–20 February 2019 through PubMed (NCBI), EMBASE, PsycINFO (Ovid), Web of Science, CINAHL (EBSCOhost), and Scopus (Elsevier). On 12–14 April 2021, the searches were updated for PubMed and Web of Science. The reference lists of all eligible studies were also consulted.

For each database, five sets of keywords were used referring to (i) population (workers); (ii) exposure (factors of the DCS and ERI models); (iii) concepts and terms that refer to both population and exposure (eg, “work stress”); (iv) outcome (synonyms for T2DM); and (v) prospective study design. The original search was not restricted by date of publication, language nor country of origin. The complete search strategy is available in the supplementary material, https://www.sjweh.fi/article/3987, table S1.

### Selection process

As a pilot, two researchers independently reviewed papers published only between 2014 and 2019. Comparison of divergences was used to clarify eligibility criteria. Using these criteria, titles and abstracts of all papers were evaluated for potential relevance. At this stage, a concordance rate (Cohen’s kappa coefficient) of 0.713 between two reviewers was calculated. Then, each of the reviewers read the full text of any publication considered by either of them to be “relevant” or “potentially relevant” based on titles and abstracts. When articles could not be accessed, efforts were made to obtain them through the Laval University library or by contacting the authors directly. During full-text screening, potentially relevant articles were read in English, Portuguese, Spanish, German or French. A final consensus decision on inclusion was made based on the full text.

### Data collection and evaluation of risk of bias

To maintain homogeneity and reliability of data extraction, a codebook with definitions of the values to be extracted was constructed. Numeric values and comments on the study population and design, the definition and prevalence of work-related stressors and of T2DM, the type of analysist – including the covariates used – and estimates of the effect measures [odds ratio (OR) or hazard ratio (HR)] with 95% confidence intervals (CI) were extracted from each eligible study by two trained and independent reviewers (table 1).

**Table 1:** Characteristics and results of the 21 studies. [BJSQ=Brief Job Stress Questionnaire; ERI=effort–reward imbalance; FPG=fasting plasma glucose; FU=follow up; JCQ=job control questionnaire; NI=not informed; OGTT=oral glucose tolerance test; PB=participation at baseline; PCE=prevalent cases excluded; PI=proportion included.]

Study and country	Population characteristics Years of FU Type of workers N analyzed / eligible N women / men	Work-related stressors Measurement time Tool Exposed fraction	Diabetes cases (%)	Analyses / Results Model PCE: Yes/No/Unclear Covariates Results
Eriksson et al, 2013 (43) Sweden (SDPP)	Baseline: 1992–1994 (men) or 1996–1998 (women) FU: 8-10 y Middle-aged workers: 4580/7949 PB: 72%, PI:80% 3205 / 2227 Mean age: 47.4 y	Exposure at baseline, JCQ Demands: 5 items Job control: 5 items Social support: 2 items 22% high strain quadrant	OGTT level: 171 cases (3%) 149 cases in N analyzed (3.2%)	Logistic regression, OR (95% CI) PCE: Yes, but N unknown Age, sex, educational level, psychological distress Demands: 0.7 (0.5–1.1) Low job control: 1.2 (0.8–1.9) Job strain tertiles: 1.6 (1.0–2.6) High vs. Low strain: 0.8 (0.5–1.3)
Garbarino et al, 2018 (34) Italy	Baseline: 2009, FU: 5y, Police (men) 234 / 294 PB: 99%, PI: 80% Mean age: 41 y	Repeated exposure JCQ: Demands: 5 items; Job control: 6 items; Social support: 6 items 89% job strain ERI: Effort: 6 items; Reward: 11 items 13% high ERI	FPG>100 mg/dL (5.6 mmol/L). 3 cases (1.3%)	Logistic regression, OR (95% CI) PCE: Yes Age, education, origin, marital status, housing and presence of offspring High combine stress: 6.36 (0.56–72.45)
Gilbert-Ouimet et al, 2021 (13) Canada (CCHS)	Baseline: 2009 FU: 13.5y, Workers: 12 896 / NI PB: 63%, PI: 95% 6148/6749 Mean age: 41 y	Exposure at baseline JCQ: Demands: 2 items; Job control: 5 items ♂ 36.3%, ♀ 26.1% all Highest job strain tertiles	Administrative data, hospital records or two physician service claims	Cox regression model, HR (95% CI) PCE: Yes Stratified by or controlled for sex, age, marital status, children under 12 in the house, born in Canada, ethnicity, living location, immigration status, survey year, self– reported, chronic diseases, work hours, interview method and activity restrictions at work. ♂ high strain: 0.93 (0.69–1.27) ♀ high strain: 1.23 (0.86–1.77)
Heraclides et al, 2009 (38) United Kingdom (Whitehall II)	Baseline: 1991–1993 FU 15y, Workers: 5895/10 308 PB: 73% (Heraclides 2012), PI 82% 1729/ 4166 Mean age: 48 y	Exposure at baseline JCQ: Demands: 4 items; Job control: 15 items; Social support: 6 items High demands: ♂53%, ♀47%, all 51% Low control: ♂44%, ♀67%, all 51% Low support: ♂33%, ♀34%, all 33% Job strain: ♂ 24%, ♀30%, all 25% Isostrain: ♂ 11%, ♀14%, all 12%	OGTT level and self-report 308 cases (5.2%)	Cox regression model, HR (95% CI) PCE: Yes Stratified by sex, controlled by age Demands: ♂106/2.222, 0.82 (0.63–1.07) ♀: 40/756, 1.06 (0.70–1.62) All: 146/2.978, 0.88 (0.70–1.10) Job control: ♂: 84/1846, 0.86 (0.66–1.13) ♀: 63/1171, 1.09 (0.70–1.69) All: 147/3017, 0.94 (0.75–1.18) Social support: ♂:69/1377, 1.0 (0.75–1.33), ♀: 31/585, 1.08 (0.70–1.67) All: 100/1962, 1.02 (0.81–1.30) Job strain: ♂: 43/987, 0.82 (0.59–1.15) ♀: 35/512, 1.59 (1.03–2.45) All: 78/1499, 1.04 (0.80–1.34) Iso–strain: ♂: 25/475, 1.07 (0.71–1.63) ♀: 20/24, 1.94 (1.17–3.21) All: 45/716, 1.33 (0.97–1.83)
Heraclides et al, 2012 (39) United Kingdom (Whitehall II)	Baseline: 1991–1993 FU: 18y Workers: 5138 /10308 PB: 73%, PI 72% 1449/3689 Mean age: 49y	Exposure at baseline JCQ Demands: 4 items Job control: 15 items Job strain: ⫿25%, ⫿32%, all 27%	OGTT level and self-report 927 cases (18%)	Cox regression model, HR (95% CI) PCE: Yes Stratified by sex, controlled by age, employment grade, diet pattern, alcohol consumption, physical activity, smoking status, systolic blood pressure, triglycerides, high–density lipoprotein cholesterol ♂: 389/3.689, high strain: 0.80 (0.63–1.02) ♀: 151/1.449, high strain: 1.37 (0.98–1.92)
Hino et al, 2016 (32) Kanto Japan	Baseline: 2008 and 2011 FU: 3y Male workers: 1815 / 29 586 PB: 21%, PI 43% Age: ≥35y	2 waves exposure: BJSQ Demands: 3 items Job control: 3 items Social support: 3 items each colleagues resp. supervisor: Demand increase: 9% Control increase: 14% SS supervisor incr.: 12% Coll. support incr.: 12% Job strain incr.: 9%	FPG, HbA1c, and immuno-reactive insulin (IRI) levels (≥2.5 on HOMA-IR) 136 cases (7.5%)	Logistic regression, OR (95% CI) PCE: Yes Age, marital status, occupational characteristics (job department, employment position and occupation Demands increase: 0.45 (0.19–1.03) Job control increase: 0.59 (0.31–1.12) Colleagues support increase: 0.86 (0.48–1.56) Supervisor support increase: 0.65 (0.33–1.28) Job strain increase: 0.56 (0.25–1.22)
Huth et al, 2014 (30) Germany (MONIKA/KORA)	Baseline: 1984-1994 FU: 12,7y Workers: 5337 /17 438 PB: 75%, PI 73% 1986 / 3351 Mean age: 43.9y	Exposure at baseline Adaptation of JCQ Demands: 5 items Job control: 6 items High strain: 19%	Self-reported and the date of diagnosis validated by hospital 291 cases (5.5%)	Cox proportional hazard model, HR (95% CI) PCE: Yes Age, sex, baseline survey, education and physical intensity work Job strain: 1.24 (0.93–1.65) High vs. low strain: 1.43 (1.00–2.06)
Kawakami et al, 1999 (35) Japan (Electrical)	Baseline: 1984 FU: 8y Male workers: 2194 / 3862 PB:92%, PI:77% Range: 18-60 y	Exposure at baseline Adaptation of JCQ Demands: 1 question Job control: 1 question Support: 1 question Job strain: 10% Low support: 19%	FPG ≥110 mg/dl + oral glucose tolerance test 34 cases (1,5%)	Cox proportional hazard model, HR (95% CI) PCE: Yes Age, education, BMI, alcohol, consumption, smoking, leisure time, physical activity, family history Job strain: 1.34 (0.50–3.55) Low social support: 1.27 (0.58–2.79)
Kroenke et al, 2007 (12) USA (NHS II)	Baseline: 1993, FU 6y Women nurses: 62 574 / 116 608 PB:75%, PI: 73% Mean age: 38.8y Range: 29-46y	Exposure at baseline JCQ, 27 items High strain: 20%	Self-reported, high confirmation rate, 365 cases (5.8%)	Cox proportional hazard model, HR (95% CI) PCE: Yes Age High strain: 1.13 (0.84–1.51)
Kumari et al, 2004 (36) United Kingdom (Whitehall II)	Baseline: 1992-93, FU 5-6y White-collar workers: 8386 / 10 308 PB:73%, PI 82% 2579 / 5807	Exposure at baseline JCQ Demands: 4 questions Job control: 15 questions Support: 6 questions ERI (proxy, no information on the number of items per dimension) high effort, low reward Fraction NI	OGTT level and self-report 361 cases (4.3%)	Logistic regression, OR (95% CI) PCE: Yes age, length of follow–up, employment grade, ethnic group and ECG abnormalities Demands: ♂ 1.11 (07–1.7), ♀: 0.59 (0.3–1.2) Job control: ♂ 0,77 (0.5–1.2), ♀: 0.82 (0.4–1.6) Support: ♂ 0.80 (0.5–1.1), ♀: 1.20 (0.7–1.9) ERI highest vs lowest category: ♂ 1.71 (1.0–2.8), ♀: 0.92 (0.4–1.9)
Mortensen et al, 2017 (29) France (GAZEL) Sweden (SLOSH) United Kingdom (Whitehall II)	Baseline: 2000 (GAZEL), 2006 (SLOSH), 1991–1994 (Whitehall II) FU 10 y White-collar workers: GAZEL: 6572/ 20625 SLOSH: 7590/40877 Whitehall: 7081/10308 PB: 45% (GAZEL), ~39% (SLOSH), 73% (Whitehall II) PI: 51% (GAZEL), 46% (SLOSH), 77% (Whitehall II) 8710 / 12 533 Range: 46–55 y	Exposure at baseline JCQ Demands:5 items Job control: 6 items Support: 2 items High strain 22% Low support 36%	Self-reported complemented with OGTT and FPG 1058 cases (433; 208; 417 resp) (5.0%)	Logistic regression OR (95% CI) PCE: Yes Age, sex, marital status, occupation and sub–cohort High job strain: GAZEL: 1.24 (0.93–1.64) SLOSH: 0.96 (0.65–1.41) Whitehall: 0.93 (0.70–1.22) Low support: GAZEL: 1.07 (0.85–1.35) SLOSH: 1.26 (0.93–1.69) Whitehall: 1.27 (0.99–1.64)
Mutambudzi et al, 2016 (40) USA (HRS)	Baseline: 2006 FU: 7y Middle- and older-aged workers: 1396 / 18 469 PB: 74%, PI:19– 50% Mean age: 58 y range ≥50	Exposure at baseline JCQ-like Demands: 3 items Job control: 3 items High strain 11%	Self-reported 167 cases (11.5%)	Cox proportional hazard model, HR (95% CI) PCE: Yes Adjusted for body mass index, physical activity, education, race, gender, alcohol use, average work hours/week, occupational category, marital status, insurance coverage, and hypertension. Low strain was treated as the referent category. High strain vs low strain: 1.73 (1.09–2.75)
Mutambudzi et al, 2018 (37) USA (HRS)	Baseline: 2006 FU: 7y Middle- and older-aged workers 1932 / 18 469 PB: 74%, PI: 24–59% 1041 / 894 Mean age: 61 y range ≥50	Exposure at baseline ERI Effort: 2 items Reward: 5 items ERI 25%	Self-reported 288 cases (11.8%)	Cox proportional hazard model, HR (95% CI) PCE: Unclear Age, sex, race, education, marital status ERI: 1.18 (0.94–1.48)
Norberg et al, 2007 (33) Sweden (VIP)	Baseline: 1989-2000, FU: 12y Nested case-cohort workers 191 cases, 393 controls PB 52%, PI: NA 240/344 Range: 40 or 50 or 60 y at baseline	Exposure at baseline JCQ Demands+ Job control: 10 items High strain: 11%	Administrative data 191 cases	Logistic regression, OR (95% CI) PCE: Yes Matched by age, sex and survey year High strain vs low strain: ♂ 1.00 (0.5–2.00), ♀ 2.8 (1.1–7.6)
Nordentoft et al, 2020 (20) Denmark (WEHD)	Baseline: 2012, 2014, 2016 FU 2.7 y Active general population: 50 552 / 115 564 PB: 54%, PI 97% 26 378 / 26 378 Range: 30-64 y	Exposure at baseline ERI Effort: 6 items Reward: 5 items ERI 25%	Administrative data 347 cases (0.69%)	Cox proportional hazard model, HR (95% CI) PCE: Unclear sex, age, cohabitation, young children in the household, SES, migration background, survey year and sample method Dichotomic ERI: both sexes 1.27 (1.02–1.58) Continuous ERI: both sexes 1.09 (0.98–1.21), ♂ 1.09 (0.95–1.25), ♀ 1.08 (0.93–1.26)
Nyberg et al, 2014 (11) Europe (IPD-Work)	Baseline: 1986-2008, 13 individual studies, FU: 23y Workers: 124 808 / NI PB: 41-82%, PI: 53-98% 70802/54006 Mean age: 49 y	Exposure at baseline JCQ harmonized Demands: 2-6 items Job control: 5-6 items Job strain 16%	Depending on the individual studies: Self-reported, OGTT and administrative records 3703 cases (3.0%)	Cox proportional hazard model, HR (95% CI) PCE: Yes Age, sex, occupational title Dichotomous job strain: 1.15 (1.06–1.25) High strain *vs.* low strain (supplementary results published separately in (25)): 1.13 (1.02–1.25)
Pan et al, 2017 (16) Sweden (SNAC-K)	Baseline: 2001–2004, FU: 3 y for >78 years and 6y for <78 years old Retired workers: 2719 / 3363 PB: 73%, PI: 88% 1756/963 Mean age: 49 y	Exposure during work life JCQ-Matrix Demands: 2 items Job control: 12 items Median score for each type of occupation Job strain 21%	Self-reported or administrative data or HbA1c >6.4% 154 cases (5.7%)	Logistic regression, OR (95% CI) PCE: Yes Age, sex, educational level, follow–up time Job strain: 1.60 (1.07–2.39)
Smith et al, 2012 (31) Canada (CCHS)	Baseline: 2000-2001, FU 10y Workers 7443/NI PB: 84% NI, PI: 89.6% 3752/3691 range: 35-60	Exposure at baseline JCQ-like Demands: 2 items Job control: 5 items Support: 3 items Low demands: ♂ 23%, ♀ 18%. Low job control: ♂ 20%, ♀26%. Low support: ♂18%, ♀18%	Administrative data 639 cases (8.7%)	Cox proportional hazard model, HR (95% CI) PCE: Yes Age, immigration status, ethnicity, marital status, urban or rural living location, education, heart disease at baseline, hypertension at baseline, depression at baseline, activity limitations at work due to health problems, and other work variables (shift schedule, weeks worked, multiple jobs, physical activity at work). Low demands (4^th^ quartile): ♂: 0,72 (0.45–1.14), ♀ 0.75 (0.43–1.33), Low control (4^th^ quartile): ♂: 0,84 (0.48–1.45), ♀ 2.17 (1.23–3.83) Low support (4^th^quartile): ♂:1.19 (0.68–2.10), ♀ 0.43 (0.23–0.82)
Souza Santos et al, 2020 (19) Brazil (Elsa-Brasil)	Baseline:2008-2010, FU y Workers civil servants 7503/ 52137 PB: 29%, PI: 86% 3998/3505 Mean age: 52y range: 35-74	Exposure at baseline DCS, ERI Demands: 5 items Job control: 6 items Support: 6 items Effort: 6 items Reward: 10 items All measures for tertiles. High job strain: ♀39.1% ♂ 28.8% Low SS: ♀36.2% ♂ 30.4% Iso-strain: ♀24.9% ♂ 16.7% High effort/reward: ♀ 37.1♂ 32.6% High Overcommitment: ♀ 31.9 ♂ 29.6% High Job strain + ERI: ♀25.7% ♂ 16.7%	HbA1c ≥6.5% 167 cases (2.2%)	Logistic regression, OR (95% CI) PCE: yes Age, schooling level, weekly workload, work shift. Most unfavorable tertile vs. most favorable tertile. High demands: ♂1.22 (0.66–2.27), ♀2.41 (1.30–4.50) Low job control: ♂0.95 (0.51–1.77), ♀0.64 (0.34–1.18) High job strain: ♂1.02 (0.59–1.76), ♀1.77 (0.98–3.19) Low support: ♂1.29 (0.69–2.40), ♀1.93 (0.96–3.87) High effort: ♂1.14 0.64–2.04), ♀1.17 (0.68–2.01) Low reward: ♂0.96 (0.55–1.68), ♀1.76 (1.03–2.99) High effort/reward: ♂1.08 (0.62–1.88), ♀1.36 (0.81–2.29) High Overcommitment: ♂ 0.81(0.44–1.49), ♀ 1.46 (0.80–2.63) High Job strain + ERI: ♂ 0.92 (0.49–1.75), ♀ 2.10 (1.20–3.65)
Toker et al, 2012 (43) Israel	Baseline: 2003 and 2008 FU: 8y 5843 / 12754 PB: 92%, PI 55% NI Mean age: 48 y	Exposure at baseline JCQ-like Demands: 6 items Job control: 7 items Social support: 8 items Fractions NI	FPG ≥ 126 mg/dL or HbA1c ≥ 6.5% or Self-reported 182 cases (3,1%)	Logistic regression, OR (95% CI) PCE: Yes Age, sex, education, follow–up time, family history, LDL, body mass index, systolic pression, triglycerides, smoking, physical activity, depression High demands: 0.98 (0.83–1.15) Low job control: 1.05 (0.85–1.29) Low support: 0.79 (0.62–0.99)
Yamaguchi et al, 2018 (41) Japan (Furukawa Nutrition and Health Study)	Baseline: 2012-2013 FU: 3 y 1040/ 2828 PB: 76%, PI 56% 115/925 Mean age: 42 y Range:19-68	Repeated exposure in two waves JCQ-like Demands: 5 items Job control: 9 items Social support: 8 items Job strain increase: 16%	FPG ≥100 mg/dL 64 cases (6.8%)	Logistic regression, OR (95% CI) PCE: Unclear, exclusion of subjects with metabolic syndrome age, sex, site, family structure, marital status, occupational category, work status, night or rotating shift work, work–related physical activity, leisure–time physical activity, smoking, alcohol drinking, sleep duration, quality of sleep, energy intake, and each component of metabolic syndrome at baseline. Job strain increase: 3.86 (1.77–8.38)

To assess the strength of the evidence for each study, we applied the ROBINS-I tool (17), recently adapted by Duchaine et al (18) for prospective occupational observational studies (supplementary text 1). For each study, two independent and blinded reviewers evaluated five bias domains: (i) confounding, (ii) selection of participants into the study, (iii) classification of interventions, (iv) missing data, and (v) measurement of outcomes. In each domain, the risk of bias was graded as “low”, “moderate”, “serious” or “critical”. However, following the adapted ROBINS-I tool, the risks of confounding bias and bias for selection at study entry were never considered low (18).

Studies that used clinical criteria for diagnosing T2DM that deviated from the official American Association for Diabetes or World Health Organization guidelines (4, 24) were considered to have a critical risk of bias in measurement of outcomes.

### Meta-analysis

For meta-analyses, when studies reported estimates of risk in more than one cohort, the results for each individual cohort were used. Among different risk estimates for the same exposure measure in one cohort, we gave preference to those that reported HR and those that used dichotomized exposure. On the other hand, whenever there was more than one publication that estimated an effect from the same kind of exposure in the same cohort, we used only the one that had the highest average follow-up or the most collection waves. The estimates for job strain by quadrants published in a note by Kivimäki et al (25) were evaluated together with the original publication (11). For each exposure scale, the choice of the most appropriate model was made based on adjustment at least for sex, age and socio-economic status (eg, education, occupation type, income), and the absence of adjustment for possible mediating variables.

Results are shown for whole populations and male and female subjects separately. When incidence estimates were available, HR and OR were transformed to rate ratios (RR) (26), but only if this could be done for all cohorts in a given meta-analysis. In order to convert HR into RR, it was necessary to first estimate *r*_0_, the rate of incidence of diabetes among the non-exposed, from the overall rate of incidence of diabetes *r*, from the proportion of unexposed subjects, *p*_0,_ and exposed subjects, *p*_1_, and from the HR as

*r*_0_=*r* / (*p*_0_ + *p*_1_^⋅^ HR)

RR were then estimated as:

RR=(1-e^HR ⋅ ln(1-*r*0)^) / *r*_0_

Analogously, the cumulative incidence of cases of diabetes at the end of a study, *f*_0_, was estimated from the overall cumulative incidence, *f*, as

*f*_0_=*f* / (*p*_0_ + *p*_1_^⋅^ OR).

RR were then estimated from odds ratios (OR) as

RR=OR / (1 – *f*_0_ + *f*_0_^⋅^ OR)

For each form of exposure, we estimated its combined effect on the incidence of T2DM from the individual studies using random effects meta-analyses and assessed heterogeneity with the I^2^ statistic and the Cochran Q-test (τ^2^). Supplementary analyses were done on subgroups defined by effect measure and by overall risk of bias. All calculations were performed using the R library meta (27, 28) at a significance level of P<0.05. Forest plots and funnel plots were generated by the same library.

## Results

We found a total of 2479 potentially eligible citations, and 113 studies were retained for full text reading (supplementary figure S1). Finally, data from the 21 publications that met our criteria were extracted for systematic review (table 1), quality assessment (table 2) and meta-analysis.

**Table 2:** Quality evaluation of prospective studies on psychosocial work factors and diabetes according to Risk Of Bias In Non-randomised Studies-intervention tool (ROBINS-I adapted) criteria. [DC=demand–control; ERI=effort–reward imbalance

Study	Risk of bias due to confounding	Bias in selection of participants into the study	Bias in classification of exposure	Risk of bias due to missing data	Risk of bias in measurement of outcomes	Highest risk of bias
Eriksson et al, 2013 (42)	Serious	Serious	Moderate	Moderate	Low	Serious
Garbarino et al, 2018 (34)	Serious	Moderate	Serious	Moderate	Critical	Critical
Gilbert-Ouimet et al, 2021 (13)	Serious	Serious	Serious	Low	Low	Serious
Heraclides et al, 2009 (38)	Serious	Serious	Moderate	Moderate	Moderate	Serious
Heraclides et al, 2012 (39)	Serious	Serious	Moderate	Serious	Moderate	Serious
Hino et al, 2016 (32)	Moderate	Critical	Moderate	Critical	Critical	Critical
Huth et al, 2014 (30)	Moderate	Serious	Low	Serious	Moderate	Serious
Kawakami et al, 1999 (44)	Serious	Moderate	Serious	Serious	Moderate	Serious
Kroenke et al, 2007 (12)	Moderate	Serious	Low	Serious	Moderate	Serious
Kumari et al, 2004 (36)	Serious	Serious	DC (moderate); ERI: (serious)	Moderate	Moderate	Serious
Mortensen et al, 2017 (29) - GAZEL	Moderate	Critical	DC (low), social support (serious)	Critical	Serious	Critical
Mortensen et al, 2017 (29) - SLOSH	Moderate	Critical	DC (low), social support (serious)	Critical	Moderate	Critical
Mortensen et al, 2017 (29) -Whitehall	Moderate	Serious	DC (moderate), social support (serious)	Serious	Moderate	Serious
Mutambudzi et al, 2016 (40)	Critical	Serious	Serious	Critical	Serious	Critical
Mutambudzi et al, 2018 (37)	Moderate	Serious	Serious	Critical	Serious	Critical
Norberg et al, 2007 (33)	Serious	Critical	Low	Moderate	Low	Critical
Nordentoft et al, 2020 (20)	Moderate	Serious	Moderate	Low	Low	Serious
Nyberg et al, 2014 (11) -COPSOQ-I	Moderate	Serious	Serious	Low	Low	Serious
Nyberg et al, 2014 (11) -COPSOQ-II	Moderate	Critical	Serious	Moderate	Moderate	Critical
Nyberg et al, 2014 (11) -DWECS	Moderate	Serious	Serious	Low	Low	Serious
Nyberg et al, 2014 (11) - FPS	Moderate	Serious	Serious	Low	Moderate	Serious
Nyberg et al, 2014 (11) - GAZEL	Moderate	Critical	Low	Serious	Serious	Critical
Nyberg et al, 2014 (11) -HeSSup	Moderate	Critical	Low	Serious	Moderate	Critical
Nyberg et al, 2014 (11) - IPAW	Moderate	Serious	Serious	Low	Moderate	Serious
Nyberg et al, 2014 (11) -PUMA	Moderate	Serious	Serious	Low	Low	Serious
Nyberg et al, 2014 (11) -SLOSH	Moderate	Critical	Low	Serious	Moderate	Critical
Nyberg et al, 2014 (11) - Still Working	Moderate	Serious	Serious	Low	Low	Serious
Nyberg et al, 2014 (11) -Whitehall II	Moderate	Serious	Moderate	Moderate	Moderate	Serious
Nyberg et al, 2014 (11) - WOLF N	Moderate	Moderate	Low	Low	Moderate	Moderate
Nyberg et al, 2014 (11) - WOLF S	Moderate	Moderate	Low	Low	Moderate	Moderate
Pan et al, 2017 (16)	Moderate	Serious	Serious	Moderate	Moderate	Serious
Smith et al, 2012 (31)	Serious	Moderate	Serious	Moderate	Low	Serious
Souza Santos et al, 2020 (19)	Serious	Critical	Low	Moderate	Low	Critical
Toker et al, 2012 (43)	Serious	Moderate	Moderate	Serious	Moderate	Serious
Yamaguchi et al, 2018 (41)	Serious	Serious	Moderate	Serious	Critical	Critical

### Systematic review

Regarding population characteristics, almost all studies were conducted in high-income countries, with one exception (Brazil) (19). In addition to the populations of large, well-known cohorts, such as GAZEL, Whitehall II, SLOSH (11, 29), MONIKA (30), CCHS (13, 31), among others, this review also included a variety of cohorts composed of healthcare workers (12, 32, 33), police officers (34), factory workers (35), civil servants (19, 36), and workers >60 years (16). The average age of participants at baseline was 35–73 years and mean follow-up durations were 2.7–13.5 years.

Among the 21 studies included in the systematic review, 19 measured work-related stressors according to the DCS. The ERI model was included in 3 studies (19, 34, 36), while 2 defined work-related stressors exclusively according to the ERI model (20, 37). Within the DCS framework, 14 studies used categorical or continuous job strain as exposure (11–13, 16, 19, 29, 30, 32–35, 38–41), 7 used high psychosocial demands and low control (19, 31, 32, 36, 38, 42, 43) and 8 used low social support at work (19, 29, 31, 32, 36, 38, 43, 44). Iso-strain was used in 2 studies (19, 38). Within the ERI framework, all 4 studies used categorical or continuous ERI as exposure, and only 1 (19) also used overcommitment.

T2DM was defined by three types of measures: (i) diagnosed by clinical tests of fasting plasma glucose, glucose tolerance or glycosylated hemoglobin (19, 32, 34, 41, 42); (ii) health system administrative records (13, 20, 31, 33); or (iii) self-reported through questions like “Has a doctor ever told you that you have diabetes or high blood sugar?” (37, 40). Nine studies used combinations of these three measurements (11, 12, 29, 30, 36, 38, 39, 43, 45). While all studies excluded prevalent cases from analyses, in one study exclusion was based on metabolic syndrome, not T2DM (41).

### Quality assessment

We assessed the quality of evidence according to the five criteria of ROBINS-I (17). Summaries of the evaluation of each study in each domain can be found in supplementary text 2.

Regarding confounding bias, 18 out of 21 studies adjusted for the potential confounding covariables sex, age and some indicators of social status. However, only 8 studies were classified as having the lowest possible risk of confounding bias for observational studies (moderate) (11, 12, 16, 20, 29, 30, 32, 37), since the others additionally adjusted for variables that potentially mediate the association between work-related stressors and T2DM (13, 19, 31, 36, 39–44).

With respect to the selection of participants, six cohorts in five studies were considered to have a moderate risk of bias (80–100% participation rate) (11, 31, 34, 35, 43). Twenty estimates were classified at serious (54–76% participation rate) (11–13, 16, 20, 29, 30, 36, 38, 39, 41, 42) and nine at critical risk of selection bias (21–59% participation rate) (11, 19, 29, 32, 33).

With regard to work-related stressors, 11 cohorts in six studies had a low risk of exposure classification bias because they used validated instruments (11, 12, 19, 29, 30, 33), while the others used instruments that had not been validated or only partially validated.

Regarding the outcome, the cohorts in most studies had a low-to-moderate risk of bias. Four cohorts had serious risk (11, 29, 37, 40), and in three studies (32, 34, 41), there was a critical risk of misclassification: defining diabetes at a cut-off of 100 mg/dl (5.6 mmol/L) fasting plasma glucose would also include patients with pre-diabetes and insulin resistance.

Regarding missing data bias, 20 cohorts in 11 studies had a low-to-moderate risk of bias, ie, 1–20% missing data (11, 13, 16, 19, 20, 31, 33, 34, 36, 38, 42).

Altogether and according to the definition of the adapted ROBINS-I tool (table 2), for 2 of the 35 published estimates, the highest risk of bias in any domain was moderate and for 20, it was serious. For the other 13 estimates, the highest risk was critical.

### Results of meta-analyses for job strain

**Figure 1:**
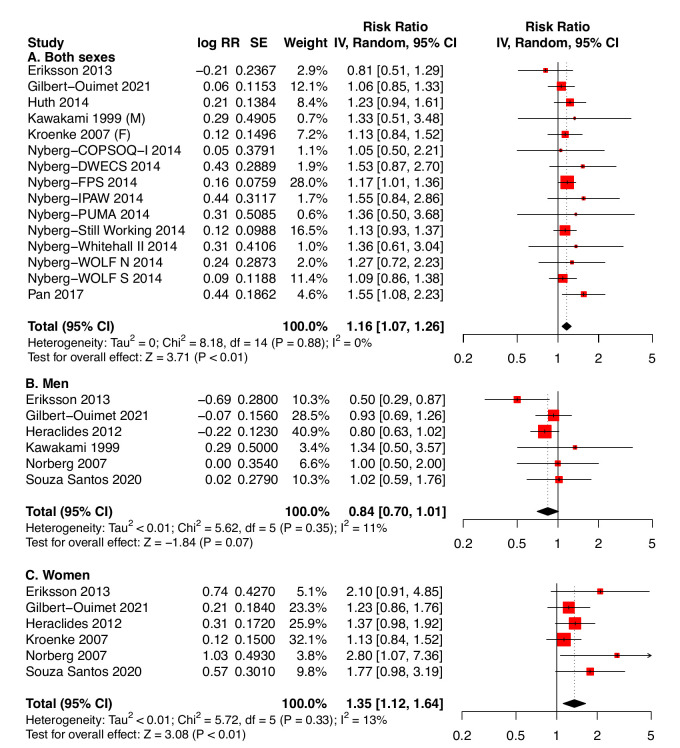
Effect of job strain on type 2 diabetes mellitus. This analysis considers job strain, whether defined as dichotomous variable (D) or as contrast between high strain and low strain quadrants (Q) and including the objective job strain matrix of Pan et al. (2017); preference was given to dichotomous job strain where available. All effect measures (OR or HR) were transformed into rate ratios. Male and female subjects in Norberg et al. (2007) were considered separately. For the Gazel, SLOSH and Whitehall II cohorts, only the estimate in Nyberg et al. (2014), which was based on the longest follow-up time, was retained. (A) Both sexes; only cohorts without critical risk of bias were included. (B) Men only. (C) Women only. SE: standard error. CI: confidence interval at 95%.

**Figure 2:**
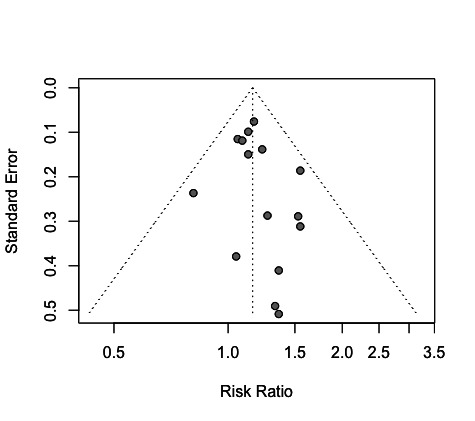
Funnel plot for the effect of job strain on type 2 diabetes mellitus. For each cohort represented in Fig. 1A, the rate ratio is plotted against its standard error. Vertical dashed line: combined rate ratio estimates from Fig. 1A. The distribution is approximately symmetric, suggesting an absence of strong publication bias.

Altogether, 28 cohorts from 21 studies met our inclusion criteria. Meta-analyses were performed whenever there were at least three independent risk estimates for the same type of exposure measure; therefore, 25 cohorts from 18 studies were used in meta-analyses. For job strain, we used the individual estimates from each of the 15 cohorts that had no critical risk of bias in any domain (figure 1A). Estimates published by Heraclides et al (38), Heraclides et al (39) and Mortensen et al (29) refer to populations also reported by Nyberg et al (11), but with shorter follow-up durations. They were therefore disregarded. According to the summary estimate, workers exposed to job strain had a higher risk of T2DM compared to non-exposed workers: RR 1.16 (95% CI 1.07-1.26). There was no evidence of heterogeneity between studies (I^2^=0%, τ^2^=0; P=0.88). The funnel plot shows no outlier studies (figure 2), and there is no statistical evidence for a publication bias in this set (P=0.27).

In subgroup analyses (figure 1B-C), women exposed to job strain had a higher pooled estimate (RR 1.35), but, due to the smaller number of studies (12, 13, 19, 33, 39, 42), the CI included most of the estimate range for the total population (95% CI 1.12–1.64). There was low heterogeneity (I^2^=13%, τ^2^ < 0.01; P=0.33). For men, there was a tendency towards an apparent protective effect of job strain against T2DM incidence [RR 0.84 (95% CI 0.70–1.01), I^2^=11%, τ^2^ < 0.01, P=0.35], but this tendency disappeared in supplementary analyses (see Section 3.5). When comparing only results from those cohorts that published results for both sexes separately (13, 19, 33, 39, 42), the difference between the effects in the strata of sex was significant (χ^2^=15.12, df=1, P<0.01).

High psychosocial demands, low control and low social support at work were not significantly associated with the incidence of T2DM in the whole population nor in strata of sex (supplementary figure S2-4). However, job control showed a tendency towards a protective effect against T2DM in men (supplementary figure S3B).

### Results of meta-analyses for ERI

**Figure 3:**
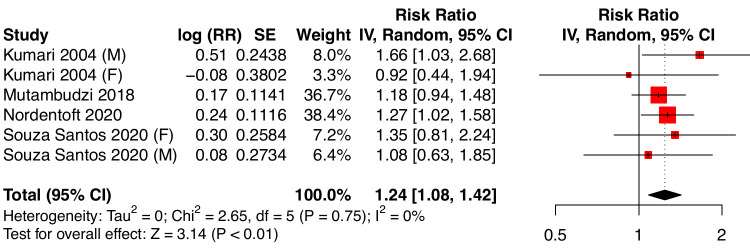
Effect of effort-reward imbalance on type 2 diabetes mellitus. Male and female subjects were considered separately. All effect measures (OR or HR) were transformed into rate ratios. SE: standard error. CI: confidence interval at 95%.

All analyses up this point refer to the DCS model, which has been the subject of previous meta-analyses. Pooled effects for the other major model for work-related stressors, the ERI model, had not yet been estimated because there were not enough original studies available. Our analysis of four studies, including two very recent ones (figure 3) (19, 20, 36, 37) indicates a significant pooled effect [RR 1.24 (95% CI 1.08–1.42); I^2^=0%, τ^2^ < 0.01, P=0.75]. These estimates are based on categorical definitions of ERI (table 1). There are not yet enough sex-specific estimates for categorical ERI to allow subgroup analyses.

### Supplementary analyses

We performed a supplementary meta-analysis for all 22 cohorts that analyzed the effect of job strain, irrespective of their risk of bias, and using the published HR and OR values without transformation (supplementary figure S5). The estimate was very similar to the main analysis [relative risk 1.17 (95% CI 1.09–1.25); I^2^=0%; τ^2^ < 0.01, P=0.79].

We also repeated this analysis using the pooled estimate previously published by Nyberg et al (supplementary figure S6). The point estimate and CI are slightly higher than in our main analysis [RR 1.19 (95% CI 1.07–1.33)], but there was evidence of moderate heterogeneity (I^2^=22%; τ^2^ <0.01, P=0.23) and of possible publication bias in this supplementary analysis (supplementary figure S7). The effects estimated separately for women [RR 1.26 (95% CI 1.05–1.51); I^2^=32%; τ^2^=0.01, P=0.20] and men [RR 0.97 (95% CI 0.75–1.25); I^2^=55%; τ^2^=0.05, P=0.05] broadly maintained the same relation as observed in the main analysis, but suffered from higher heterogeneity.

In all analyses up to this point, we combined studies that defined job strain as a continuous variable, as a dichotomous variable (job strain versus no strain) or as job strain quadrants (high strain versus low strain). In another supplementary analysis, we separated these study groups. For dichotomous exposure, we estimated RR 1.16 (95% CI 1.07–1.26; I^2^=0%; τ^2^=0%, P= 1.00). In the case of job strain quadrants, we estimated RR 1.21 (95% CI 1.02–1.43; I^2^=40%; τ^2^=0.02, P=0.12). The only study using continuous job strain reported RR 1.06 (95% CI 0.85–1.33).

As a final supplementary meta-analysis for the effect of ERI, we used the published HR and OR values without transformation (supplementary figure S8). Here also, the estimate was very similar to the main analysis [relative risk 1.24 (95% CI 1.08–1.43); I^2^=0%; τ^2^=0, P=0.75].

## Discussion

The main objective of this systematic review and meta-analysis, which includes 21 prospective studies with 334 132 workers and 10 806 cases of T2DM, was to synthesize the evidence regarding effects of work-related stressors (job strain and ERI) on the incidence of T2DM.

### Demand-control model

Job strain was significantly associated with the risk of developing T2DM. The risk of diabetes was 16% higher among workers exposed to job strain when compared to unexposed workers, and the magnitude and significance of this relative risk were preserved in several supplementary analyses.

Among women exposed to job strain, the risk of developing T2DM was 35% higher when compared to unexposed women, which is a significantly stronger effect than among men. The absence of an association in men might be explained by pathophysiological characteristics that make men more vulnerable to the incidence of T2DM and may eclipse the effect of work stressors. In fact, a somewhat higher overall incidence in men has been reported in the literature (46, 47), and is also present in those studies in our meta-analysis that reported cumulative incidence separately for men (6.2%) and women (4.5%). As examples of pathophysiological characteristics, men have a higher turnover of fatty acids, the accumulation of visceral adipose tissue is higher among them, and it is a risk factor for T2DM independent of total BMI (46).

Another explanation would be that men are more often employed for strenuous work than women. The protection afforded by the physical exercise involved in such jobs might overcome the higher risk of T2DM expected from high psychosocial stressors (48). Such an effect might explain the tendencies towards an inversion of the association with T2DM that we observed for both job strain and job control

Not all groups have published sex-specific effect estimates for job strain, and only a very reduced number of studies have done so for job control. To understand the direction and magnitude of associations in men and women, we suggest that the analyses of work-related psychosocial stressors, including their dimensions, that are shown here be published also stratified by sex, and we extend this suggestion to future publications.

The two most recent meta-analyses (14, 15) come to different conclusions with regard to the significance of job strain for T2DM in the total population; both of them are strongly driven by a single aggregated cohort study (11). In addition to including two recent studies (13, 19), we have here detected varying levels of risk bias among the cohorts reported in combined studies, namely those of Nyberg et al (11) and Mortensen et al (29). While our final results for job strain closely agree with those of Li et al (14), the present analysis is built on a separate evaluation of risk of bias for each cohort and avoids combined estimates; such estimates would count participants from separately published studies more than once (29, 38, 39).

Based on the few studies that evaluated high psychological demands, low control or social support, we found no significant association between any of these dimensions taken alone and the incidence of T2DM.

### Effort–reward imbalance model

The present meta-analysis is the first one to estimate the contribution of work-related stressors, as defined by the ERI model, to the risk of T2DM. Based on four studies, workers experiencing ERI have a 24% higher risk of developing T2DM than unexposed workers. When restricted to categorical definitions of ERI, only two of the studies published sex-specific effect estimates, one of which observed a tendency for a stronger effect in women (19), similar to the pooled effects we have described above for job strain, while the other one found a stronger effect in men (36). We note that a third study, using a continuous definition of ERI, found almost identical effects for men and for women (20).

### Limitations

In spite of our stringent evaluation of quality, it is necessary to consider possible remaining sources of bias. First, a confounding bias due to the presence of unmeasured factors associated both with T2DM and with work-related stressors might lead to an overestimation of the true effect. For example, poor general health may, on the one hand, induce a negative perception of the work environment and, on the other hand, increase the risk of T2DM by inducing lack of self-care and the adoption of unhealthy habits. Unmeasured symptoms of a pre-clinical diabetic state might induce a similar negative perception. However, pre-clinical diabetes develops almost imperceptibly (1), and one would therefore not expect it to impact psychosocial work stressors. In fact, if the studies had excluded or controlled for lifestyle habits, general health or pre-clinical T2DM, they might block a causal path and underestimate the total causal effects. Therefore, while all estimates included in our main analysis were adjusted for an indicator of socioeconomic status in addition to age and sex, reducing a possible confounding bias, we avoided estimates that had been adjusted for potential mediators.

Differential misclassification might also theoretically lead to overestimation of the true effect if self-reported T2DM was skewed by the stressors that the participant suffers at work. However, the cohorts included in the main job strain analysis used self-report data only in combination with clinical tests. We also consider the risk of differential misclassification of work-related stressors to be low, given that prevalent cases of T2DM were excluded from all cohorts.

Conversely, our estimates might underestimate the true effects. Non-differential misclassification of the outcome might be a problem since some of the included studies did not differentiate types of diabetes. However, since prevalent cases of type 1 diabetes at baseline were excluded, while the incidence of T2DM in adults is several orders of magnitude greater than that of type 1 (49, 50), the risk of misclassification for the outcome is quite low. The assessment of diabetes from medication reimbursement data might also misclassify some cases since some anti-diabetic medications are also used for treating other diseases. However, no study was based only on medication reimbursement data, and only one study in the main job strain analysis (figure 1A) and one in the ERI analysis (figure 3) relied in part on such data [Still Working (11) for job strain, Nordentoft et al (20) for ERI]. Therefore, it seems unlikely that the overall result of the meta-analysis would be noticeably affected.

Some studies assessed exposure using questionnaires that were not validated for the local language, with short and non-validated versions or with matrices of median scores derived from colleagues or peers of the participant. Such non-differential misclassification of exposures is expected to bias the effect estimates towards the null value.

Similarly, a healthy worker survival bias is inherent to occupational studies: workers generally have lower overall disease incidence than the general population due to the tendency for workers of ill health to be excluded from employment. Moreover, workers that are more exposed to work-related stressors may quit or change jobs in order to reduce their exposure. It is particularly difficult to exclude a healthy worker survival bias when participant characteristics were only measured at recruitment, as is the case for most of the cohorts included here. However, it would generally underestimate the true effect (51).

Finally, bias due to differential self-selection of participants at recruitment and especially to differential loss to follow-up is frequent in prospective studies. Here, evaluating the direction and magnitude of the distortion would require an in-depth knowledge of each cohort. A study has found that low initial participation rate may have a limited impact on estimates for some exposure-outcome relations (52). Among the studies included here, only one (20) has estimated the bias caused by a middling initial participation rate (54%) in the relation between work-related stressors and diabetes (53).

As such, we made the choice of having somewhat severe criteria for evaluating the risk of bias of initial participation selection bias. While we might have overestimated the true risk of bias of some studies, this procedure has mitigated the possible impact of a selection bias on our estimates.

### Strengths

A major strength of this review and meta-analysis is the extensive search across six different databases to identify all publications on work-related stressors and diabetes published from 1979 to 2021. A rigorous assessment of the risk of bias was performed for each individual cohort, which resulted in low heterogeneity between the studies. This cohort-level analysis also allowed us to exclude duplicate risk estimates from the same population that had been published in different studies. Estimation was further improved by the use of RR. Finally, several supplementary analyses using the published pooled estimates, or restricted to subsets, such as studies using dichotomous job strain or job strain quadrants, provided similar estimates as in the main results.

### Perspectives

Despite efforts to identify all eligible studies without geographical restriction, the main focus is on European, mainly Nordic, and North American cohorts. While this does not affect the internal validity of our results, they may not easily be generalizable to other regions.

The main target group of the studies was <60 years, but we can hypothesize that the effect of work-related stressors on the incidence of T2DM persists at older ages (16, 37, 40). If this is the case, since the overall incidence of T2DM is ~six times higher at age 70 then at age 40 (49), the absolute risk difference due to work-related stressors might rise if the same cohorts are accompanied until after retirement.

To put our results in perspective, it is worth comparing them with those of more proximal risk factors for diabetes, ie, factors that are widely recognized and included in worldwide programs for the prevention of chronic non-communicable diseases (3, 45). As an example of the effect of moderate physical activity, a meta-analysis concluded that among those participants who walked least, the incidence of T2DM was 18% (95% CI 9–26%) higher than among those who walked most (54). With regard to diet, subjects with high consumption of sweetened beverage had a 26% (95% CI 12–41%) higher incidence of T2DM (55). Among heavy smokers it was 28% (95% CI 4–58%) higher than among non-smokers (56). Considering that approximately 20% of workers are exposed to work-related stressors with relative risks comparable to those of such lifestyle patterns, it seems to be relevant to investigate interventions in the work environment for prevention (57).

### Concluding remarks

This systematic review and meta-analysis found evidence that workers exposed to job strain or ERI are at increased risk of developing T2DM, with women being at particularly high risk.

Our findings may be useful both for clinical practice and for conducting new research, considering that the onset of T2DM is estimated to occur 4–7 years before its clinical diagnosis (58, 59). Health professionals should be aware that patients, particularly women, who report stress at work may be at increased risk of T2DM. Considering these stressors in early screening may contribute to improve the prevention of T2DM among women.

Moving forward, more longitudinal research is needed to better assess the contribution of ERI on the risk of T2DM and whether it is modified by sex. We also suggest studies to better understand the mechanisms underlying the different effect sizes of job strain in men and women, and studies that estimate a potential cumulative effect of work-related stressors throughout working life and into retirement.

### Conflict of interest & funding

Authors declare no conflicts of interest.

This work was supported by the Canadian Institutes of Health Research (grant number MOP-119280); and the Fonds de recherche du Québec – Santé. The funding sources had no role in the design and conduct of the study; collection, management, analysis, and interpretation of the data; preparation, review, or approval of the manuscript; and decision to submit the manuscript for publication.

## Supplementary material

Supplementary material
