# Supplementary material for: Job strain and effort–reward imbalance as risk factors for type 2 diabetes mellitus: A systematic review and meta-analysis of prospective studies
Source: Scand J Work Environ Health. 2021 Dec 30;48(1):5–20. doi: 10.5271/sjweh.3987 (PMC8729162; doi:10.5271/sjweh.3987)
Supplement: Supplementary material [file SJWEH-48-5-S001.pdf]

Job strain and effort-reward imbalance as risk factors for type 2 diabetes mellitus: A systematic review and meta-analysis of prospective studies[1]

by Ana Paula B Pena-Gralle, MSc,[2] Denis Talbot, PhD, Caroline S Duchaine, MSc, Mathilde Lavigne-Robichaud, MSc, Xavier Trudel, PhD, Karine Aubé, MSc, Matthias Gralle, PhD, Mahée Gilbert-Ouimet, PhD, Alain Milot, PhD, Chantal Brisson, PhD

1. *Supplementary Material*
2. *Correspondence to: Ana Paula Bruno Pena-Gralle, CHU de Québec Research Center, Population Health and Optimal Health Practices Unit, Saint-Sacrément Hospital, 1050, Chemin Ste-Foy, Québec, QC, Canada G1S 4L8. [E-mail: ana-paula.bruno-pena-gralle.1@ulaval.ca]*

Supplementary Table S1. Search strategy for PubMed.

No.	Query	
**#1**	((job [tiab] AND (control [tiab] OR security [tiab] OR insecurity [tiab] OR strain [tiab] OR stress [tiab] OR stressor [tiab] OR stressors [tiab] OR demand [tiab] OR demands [tiab] OR demanding [tiab])))	**WORK RELATED STRESSORS**
**#2**	(workload[mesh] OR workload[tiab])	
**#3**	((work [tiab] AND (stress [tiab] OR stressor [tiab] OR stressors [tiab])))	
**#4**	(#1 OR #2 OR #3)	
**#5**	(employment[mesh] OR occupations[mesh])	**WORK**
**#6**	employee*[tiab]	
**#7**	((job [tiab] OR jobs [tiab]))	
**#8**	employment[tiab]	
**#9**	work[ti]	
**#10**	work[mesh]	
**#11**	workplace[mesh]	
**#12**	((occupation [tiab] OR occupations[tiab)])	
**#13**	workplace*[tiab]	
**#14**	(#5 OR #6 OR #7 OR #8 OR #9 OR #10 OR #11 OR #12 OR #13)	
**#15**	sociological factors[mesh]	**STRESSORS**
**#16**	stress, psychological[mesh]	
**#17**	social justice[mesh]	
**#18**	(reward[mesh] OR reward[tiab])	
**#19**	overcommitment[tiab]	
**#20**	(siegrist*[tiab] OR siegrist j[au])	
**#21**	(karasek*[tiab] OR karasek r[au] OR karasek ra[au])	
**#22**	skill discretion[tiab]	
**#23**	(social support[tiab] OR Social Support[mesh])	
**#24**	((iso-strain [tiab] OR iso strain [tiab]))	
**#25**	decision authority[tiab]	

No.	Query	
#26	(decision making[tiab] OR Decision Making[mesh])	
#27	((decision [tiab] AND latitude*[tiab]))	
#28	((demand* [tiab] AND latitude*[tiab]))	
#29	((demand* [tiab] AND control[tiab]))	
#30	((psychological* [tiab] AND demand*[tiab]))	
#31	((equity [tiab] OR inequities [tiab] OR inequity[tiab]))	
#32	((intrinsic [tiab] AND effort*[tiab]))	
#33	psychosocial[tiab]	
#34	(((organizational [tiab] OR organisational [tiab] OR distributive [tiab] OR procedural [tiab] OR interactional [tiab] OR relational [tiab]) AND (justice [tiab] OR injustice [tiab])))	
#35	(#15 OR #16 OR #17 OR #18 OR #19 OR #20 OR #21 OR #22 OR #23 OR #24 OR #25 OR #26 OR #27 OR #28 OR #29 OR #30 OR #31 OR #32 OR #33 OR #34)	
#36	diabetes mellitus, type 2[mesh]	**DIABETES**
#37	((Diabetes Mellitus [tiab] AND (Ketosis-Resistant [tiab] OR Ketosis Resistant [tiab] OR Ketosis-Resistant [tiab])))	
#38	((Diabetes Mellitus [tiab] AND (Noninsulin-Dependent [tiab] OR Non Insulin Dependent [tiab] OR Noninsulin Dependent [tiab] OR Non-Insulin-Dependent [tiab])))	
#39	((Diabetes Mellitus [tiab] AND Stable [tiab]))	
#40	((Diabetes Mellitus, Type II [tiab] OR Type 2 Diabetes [tiab] OR Diabetes, Type 2 [tiab] OR Type 2 Diabetes Mellitus [tiab]))	
#41	((Diabetes [tiab] AND (Maturity-Onset [tiab] OR Maturity Onset [tiab] OR Slow-Onset [tiab] OR Slow Onset [tiab] OR Adult-Onset [tiab] OR Adult Onset [tiab])))	
#42	NIDDM[tiab]	
#43	MODY[tiab]	
#44	(("metabolic diseases"[MeSH Terms] OR "metabolic syndrome"[MeSH Terms] OR "metabolic syndrome"[TIAB] OR "metabolic diseases"[TIAB]))	
#45	(("Impaired glucose tolerance"[TIAB]OR "insulin glucose tolerance"[TIAB]OR IGT[TIAB] OR “impaired glucose” [TIAB]))	
#46	(("impaired fasting glycaemia"[TIAB] OR Hyperglycaemia[TIAB]OR "impaired fasting glycemia"[TIAB] OR Hyperglycemia[TIAB]))	
#47	(("Insulin resistance"[TIAB] OR "insulin sensitivity"[TIAB] OR “impaired fasting insulin” [TIAB]))	
#48	((prediabetes[TIAB] OR "borderline diabetes"[TIAB] OR "subclinical diabetes"[TIAB]))	
#49	(("HOMA index"[TIAB] OR "HOMA-IR"[TiAB] OR "HOMA-β"[TIAB]))	
#50	HbA1c [TIAB] OR "glycosylated hemoglobin" [TIAB] OR "glycated hemoglobin" [TIAB] OR "glycohemoglobin" [TIAB] OR "glycosylated haemoglobin" [TIAB] OR "glycated haemoglobin” [TIAB] OR “glycohaemoglobin" [TIAB]	

No.	Query	
**#51**	(#36 OR #37 OR #38 OR #39 OR #40 OR #41 OR #42 OR #43 OR #44 OR #45 OR #46 OR #47 OR #48 OR #49 OR #50)	
**#52**	Prospective[MeSH Terms] OR Prospectively [MeSH Terms] OR cohort [MeSH Terms] OR incidence [MeSH Terms] OR incident [MeSH Terms] OR Cox [MeSH Terms] OR "hazard ratio" [MeSH Terms] OR "proportional hazards" [MeSH Terms] OR longitudinal [MeSH Terms] OR "follow up" [MeSH Terms] OR "follow-up" [MeSH Terms]	**LONGITUDINAL DESIGN**
**#53**	Prospective[TIAB] OR Prospectively [TIAB] OR cohort [TIAB] OR incidence [TIAB] OR incident [TIAB] OR Cox [TIAB] OR "hazard ratio" [TIAB] OR "proportional hazards" [TIAB] OR longitudinal [TIAB] OR "follow up" [TIAB] OR "follow-up" [TIAB]	
**#54**	(#52 OR #53)	
**#55**	(#4 AND #51)	**COMBINATIONS**
**#56**	(#14 AND #35 AND #51)	
**#57**	(#55 OR #56)	
**#58**	(#54 AND #57)	

Supplementary Text S1. The Risk Of Bias In Non-randomized Studies – of Interventions (ROBINS-I) assessment tool

(version for cohort-type studies)

Version 19 September 2016

This work is licensed under a Creative Commons Attribution-NonCommercial-NoDerivatives 4.0 International License.

ROBINS-I tool (Stage I): At protocol stage

1. Is there an effect of psychosocial stressors at work defined by the DCS and/or ERI models, on the incidence of type 2 diabetes mellitus?

Participants	Workers of all types, without diabetes at recruitment, from any country. Must not be a population of workers who are off work for illness or who are in a process of returning to work. Must not be a population comprising only pregnant women nor a population defined by any preexisting illness.
Experimental intervention	**Type:** Exposure must have been measured with a validated tool based on one of two models studied (Demand-Control or Effort-Reward imbalance). The validity must have been demonstrated in a study on the psychometric qualities of the instrument (internal consistency, factorial validity, predictive validity and discriminant validity)<br>**Frequency**: Participants must have been exposed for a sufficient amount of time to allow the appearance of the outcome. We consider one year of exposure to be enough.<br>**Consideration of past exposure:** To avoid the consideration of previous exposure, a cohort must be comprised of new workers, never exposed before. A cohort of participants who would all be exposed (or all unexposed) in which we study the change in exposure would be considered to adequately account for past exposure.<br>**Measurement time:** The exposure must have been measured at the beginning of the study, without participants knowing the nature of the study.<br>**Healthy Cohort in Exposure Measurement**: Participants should not have diabetes when measuring exposure (*i.e.* exposure evaluators are not influenced by knowledge of the outcome). Prevalent cases should be excluded.
Comparator	Exposed and unexposed workers originate from the same study population.
Outcomes	**Diabetes mellitus, type 2:**<br>• Information obtained from administrative data OR a clinical test OR diagnostic by a physician.

List the confounding domains relevant to all or most studies

**Major confounding domains** (for which we want the analyzes to be compulsorily adjusted): Socio-economic status (ideally education or income, but we also accept occupation), Age and Sex.<br>**Additional confounding domains**, but optional (we use the most adjusted model without including intermediate domains): Work Environment Factors, Family Charge, Stressful Events, Out of Work Social Support, Gender<br>**Confounding and Intermediate domains (should not be adjusted for)**: Body mass index (BMI), Lifestyle factors, Comorbidities, hours worked per week, multiple jobs

~~List co-interventions that could be different between intervention groups and that could impact on outcomes~~

Non-Applicable

Specify the outcome

Specify which outcome is being assessed for risk of bias (typically from among those earmarked for the Summary of Findings table). Specify whether this is a proposed benefit or harm of intervention.

- **Outcome: Harmful outcome:** Diabetes mellitus, type 2.
- **Administrative data** with codes ICD-9-CM 250 or ICD-10-CM E11-E14) **OR**
- **Clinical measurements** following the guidelines of the American Diabetes Association (ADA): Fasting Plasma Glucose (FPG)>=126 mg/dl or Oral Glucose Tolerance Test (OGTT) >=200 mg/dl or Hemoglobin A1c>=6.5% or symptoms plus random plasma glucose >=200 mg/dl **OR** Diabetes **diagnostic** by a physician, including **self-reported** diagnostic

Specify the numerical result being assessed

In case of multiple alternative analyses being presented, specify the numeric result (e.g. RR = 1.52 (95% CI 0.83 to 2.77) and/or a reference (e.g. to a table, figure or paragraph) that uniquely defines the result being assessed.

HR, OR, RR, Rate ratio (Poisson regression) and their respective 95% confidence intervals.

Preliminary consideration of confounders

Complete a row for each important confounding domain (i) listed in the review protocol; and (ii) relevant to the setting of this particular study, or which the study authors identified as potentially important.

"Important" confounding domains are those for which, in the context of this study, adjustment is expected to lead to a clinically important change in the estimated effect of the intervention. "Validity" refers to whether the confounding variable or variables fully measure the domain, while "reliability" refers to the precision of the measurement (more measurement error means less reliability).

**(i) Confounding domains listed in the review protocol**				
Confounding domain	Measured variable(s)	Is there evidence that controlling for this variable was unnecessary?*	Is the confounding domain measured validly and reliably by this variable (or these variables)?	OPTIONAL: Is failure to adjust for this variable (alone) expected to favour the experimental intervention or the comparator?
**MAJOR**:			Yes / No / No information	Favour experimental / Favour comparator / No information
Age	Self-reported or obtained by register, ideally as a continuous variable or categorized in at least 3 groups.	Never		
Sex / Gender	Self-reported or obtained by register	population composed solely of women or solely men		
Socioeconomic status	Education, income or occupation, self-reported or obtained by register, treated as a categorical variable (ideally at least 3 categories).	population composed of one homogenous type of work only		
**OPTIONAL**:				
Work Environment Factors				
Family Charge				

Stressful Event				
Out of Work Social Support				
Gender	Self-reported or obtained by register	If completely identical to the variable sex		
**MEDIATOR** (should not be adjusted for)				
Lifestyle habit (alcohol, smoking, physical activity				
BMI				
Comorbidity (cardiovascular disease, physical illness, self-rated health, musculoskeletal problem, etc.)				
Number of hours worked				
Multiple jobs				

* In the context of a particular study, variables can be demonstrated not to be confounders and so not included in the analysis: (a) if they are not predictive of the outcome; (b) if they are not predictive of intervention; or (c) because adjustment makes no or minimal difference to the estimated effect of the primary parameter. Note that “no statistically significant association” is not the same as “not predictive”.

Risk of bias assessment

Responses underlined in green are potential markers for low risk of bias, and responses in red are potential markers for a risk of bias. Where questions relate only to sign posts to other questions, no formatting is used.

Signalling questions	Description	Response options
**Bias due to confounding**		
1.1 Is there potential for confounding of the effect of intervention in this study?<br>~~**If N/PN to 1.1:** the study can be considered to be at low risk of bias due to confounding and no further signalling questions need to be considered~~	**YES**: The answer will always be YES in observational studies.	**Y** ~~/ PY / PN / N~~
**If Y/PY to 1.1**: determine whether there is a need to assess time-varying confounding:		
1.2. Was the analysis based on splitting participants follow-up time according to intervention received?<br>**If N/PN**, answer questions relating to baseline confounding (1.4 to 1.6)<br>**If Y/PY**, go to question 1.3.	**No:** If exposure is measured at recruitment only, or if exposure is measured at two or more times, but the authors averaged the measurement times.<br>**YES:** If the exposure is measured at several times and considered cumulatively or combined.	NA / Y / PY / PN / N / NI
1.3. Were intervention discontinuations or switches likely to be related to factors that are prognostic for the outcome?<br>~~**If N/PN**, answer questions relating to baseline confounding (1.4 to 1.6)~~<br>**If Y/PY**, answer questions relating to both baseline and time-varying confounding (1.7 and 1.8)	**IF YES at question 1.2:**<br>**YES:** Always yes. It can always be assumed that the change in exposure status may be influenced by the presence of diabetes.<br>**IF NO at question 1.2: NA**	NA / Y / PY / PN / N / NI

**Questions relating to baseline confounding only**		
1.4. Did the authors use an appropriate analysis method that controlled for all the important confounding domains?	Appropriate analyses: adjustment for predefined variables, restriction, matching, weighting.<br>Not appropriate: Forward procedure, one factor at a time in the crude model backward procedure<br>Significant domains of confounding: age, sex, socio-economic status (SES)<br>**YES**: if appropriate analysis for age, sex and SES<br>**NO**: if any of these factors were not considered or not considered appropriately in the analysis	NA / Y / PY / PN / N / NI
1.5. **If Y/PY to 1.4**: Were confounding domains that were controlled for measured validly and reliably by the variables available in this study?	Age: by register or self-reported, continuous or with at least 3 categories<br>Sex: by register or self-reported<br>SES: Self-reported for education and occupation are considered acceptable. Register for occupation is acceptable, income: self-reported suboptimal, but there is not really any other source of information possible. Therefore, we consider self-reported income acceptable. SES should be reported with at least 3 categories.<br>YES: if all three measures are relatively valid.<br>NO: if one of the measures seems very invalid or poorly measured or misclassified (e.g. age reported dichotomously)	NA / Y / PY / PN / N / NI
1.6. Did the authors control for any post-intervention variables that could have been affected by the intervention?	Potentially Intermediate Variables: BMI, lifestyle and Comorbidities, hours worked, multiple jobs<br>**NO**: if there is a model with appropriate analyzes for age, sex and SES, *without* potentially intermediate variables<br>**YES**: if there are no models with appropriate analyzes for age, sex and SES, without potentially intermediate variables	NA / Y / PY / PN / N / NI
**Questions relating to baseline and time-varying confounding**		
1.7. Did the authors use an appropriate analysis method that controlled for all the important confounding domains and for time-varying confounding?	**NO:** Any model that does not consider time-varying exposure and confounders.<br>**YES:** Marginal structural models that consider time-varying exposure and confounders (including survival models), whether exposure is operationalized as acute or cumulative.	NA / Y / PY / PN / N / NI
~~1.8. **If Y/PY to 1.7**: Were confounding domains that were controlled for measured validly and reliably by the variables available in this study?~~		

**Risk of bias judgment**	**Low:** No confounding expected: **NEVER**<br>**Moderate**: (i) Confounding expected, all known important confounding domains appropriately measured and controlled for;<br>*and*<br>(ii) Reliability and validity of measurement of important domains were sufficient, such that we do not expect serious residual confounding.<br>**Serious**: (i) At least one known important domain was not appropriately measured, or not controlled for;<br>*or*<br>(ii) Reliability or validity of measurement of an important domain was low enough that we expect serious residual confounding.<br>or<br>(iii) The authors controlled at least for some post-intervention variables that could have been affected by the intervention<br>**Critical:** (i) Confounding inherently not controllable<br>*or*<br>(ii) The use of negative controls strongly suggests unmeasured confounding.	~~Low~~ / Moderate / Serious / Critical / NI
~~Optional: What is the predicted direction of bias due to confounding?~~		~~Favours experimental / Favours comparator / Unpredictable~~

Bias in selection of participants into the study		
2.1. Was selection of participants into the study (or into the analysis) based on participant characteristics observed after the start of intervention?<br>~~**If N/PN to 2.1:** go to 2.4~~	**YES**: Always yes in our case, except with a cohort of new workers or have selected a cohort of participants who would all be exposed, or all not exposed at recruitment, and analyze the change in exposure over time.	Y / PY / ~~PN / N~~ / NI
2.2. **If Y/PY to 2.1**: Were the post-intervention variables that influenced selection likely to be associated with intervention?<br>2.3 **If Y/PY to 2.2**: Were the post-intervention variables that influenced selection likely to be influenced by the outcome or a cause of the outcome?	**YES**: Always yes, because we can expect more exposed people to leave work before the start of the study, or to participate less in the study<br>**YES**: Always yes, because we can expect more exposed people to leave work before the start of the study, or to participate less in the study.	NA / Y / PY / ~~PN / N~~ / NI
2.4. Do start of follow-up and start of intervention coincide for most participants?	**NO:** due to our field of study, this answer should always be no, except with a cohort of new workers or having selected a cohort of participants who would all be exposed or all unexposed at recruitment, and analyzed the change in exposure over time.	~~Y / PY~~ / PN / N / NI
2.5. **If Y/PY to 2.2 and 2.3~~, or N/PN to 2.4~~**: Were adjustment techniques used that are likely to correct for the presence of selection biases?	**NO:** Always no, because we never know the characteristics of the participants before the start of the study.	

**Risk of bias judgment**	**Note:** In occupational studies start of follow-up and start of exposure rarely coincide. For this reason, we choose to start the risk of bias in selection of participants into the study to moderate levels for this criterion. However, this criterion will not be considered in the other levels in order to keep a gradation in this risk of bias.<br>**Low: Never, due to the point (ii)**<br>(i) All participants who would have been eligible for the target trial were included in the study; and **(ii) For each participant start of follow up and start of intervention coincided**.<br>**Moderate:** Participation rates of **≥80% or ≥ 70%** with a comparison showing that refusals are similar to those included for age, sex and socio-economic status, or for exposure and outcome<br>(i) Selection into the study may have been related to intervention and outcome; and The authors used appropriate methods to adjust for the selection bias; or (ii) Start of follow up and start of intervention does not coincide for all participants; and (a) the proportion of participants for which this was the case was too low to induce important bias; (90% de participation) or (b) the authors used appropriate methods to adjust for the selection bias; or (c) the review authors are confident that the rate (hazard) ratio for the effect of intervention remains constant over time.<br>**Serious:** Participation rates between **80-60% or 60-50% with a comparison** showing that refusals are similar to those included for age, sex and socio-economic status, or for exposure and outcome<br>(i) Selection into the study was related (but not very strongly) to age, sex and socio-economic status or the intervention and outcome; and This could not be adjusted for in analyses; or (ii) Start of follow up and start of intervention does not coincide; and A considerable amount of follow-up time is missing from analyses; and The rate ratio is not constant over time.	~~Low~~ / Moderate / Serious / Critical / NI

	**Critical:** Participation rates of less than **<60% or <50%** with a comparison showing that refusals are similar to those included for age, sex and socio-economic status, or for exposure and outcome (i) Selection into the study was very strongly related to) to age, sex and socio-economic status or the intervention and outcome; and This could not be adjusted for in analyses; or (ii) A substantial amount of follow-up time is likely to be missing from analyses; and The rate ratio is not constant over time.	
~~Optional: What is the predicted direction of bias due to selection of participants into the study?~~		~~Favours experimental / Favours comparator / Towards null /Away from null / Unpredictable~~

Bias in classification of interventions		
3.1 Were intervention groups clearly defined?	**YES:** Exposure must have been measured by a validated tool based on one of two models studied. The validity must have been demonstrated in a study on the psychometric qualities of the instrument (internal consistency, factorial validity, predictive validity and discriminant validity). Note: If the tool used is an original validated tool, but the translation has not been validated, it is considered to be a well-defined intervention, but with a moderate level of risk.<br>**NO:** Exposure measured with a proxy or translation whose validation has not been demonstrated, or by using different questionnaires from one participant to another. Exposure measured by a matrix based on job titles or based on the response of colleagues in the same work unit, as there is a risk of significant misclassification.	Y / PY / PN / N / NI
3.2 Was the information used to define intervention groups recorded at the start of the intervention?	**YES**: if exposure is measured at beginning of follow-up<br>**NO**: if exposure is measured retrospectively	Y / PY / PN / N / NI
3.3 Could classification of intervention status have been affected by knowledge of the outcome or risk of the outcome?	**NO:** If prevailing cases were excluded<br>**PY:** If the analyzes are adjusted for diabetes or high glucose or metabolic syndrome at recruitment only without the exclusion of prevalent cases<br>**YES:** if prevalent cases are not excluded and no sensitivity analysis was conducted according to the mental health status at recruitment. In this case, the disease may have affected the response to the exposure questions.	Y / PY / PN / N / NI

**Risk of bias judgment**	**Low :** (i) Intervention status is well defined; *and*<br>(ii) Intervention definition is based solely on information collected at the time of intervention.<br>**Moderate:** (i) Intervention status is well defined; (Note: here we included the use of validated questionnaires, but without validation of the translation)<br>*and*<br>(ii) Some aspects of the assignments of intervention status were determined retrospectively.<br>**Serious:** (i) Intervention status is not well defined;<br>*or*<br>(ii) Major aspects of the assignments of intervention status were determined in a way that could have been affected by knowledge of the outcome.<br>**Critical:** (Unusual) An extremely high amount of misclassification of intervention status, e.g. because of unusually strong recall biases.	Low / Moderate / Serious / Critical / NI
~~Optional: What is the predicted direction of bias due to classification of interventions?~~		~~Favours experimental / Favours comparator / Towards null /Away from null / Unpredictable~~

**Bias due to deviations from intended interventions: NA: Hard to apply in our field of research. Exposure deviations are almost always natural and expected, unless there is an intervention by a researcher that is differential depending on the level of exposure. This criterion will always be at a moderate level of risk. Therefore, it is not systematically evaluated in the included studies.**		
~~**If your aim for this study is to assess the effect of assignment to intervention, answer questions 4.1 and 4.2**~~		
~~4.1. Were there deviations from the intended intervention beyond what would be expected in usual practice?~~		~~Y / PY / PN / N / NI~~
~~4.2. **If Y/PY to 4.1**: Were these deviations from intended intervention unbalanced between groups *and* likely to have affected the outcome?~~		~~NA / Y / PY / PN / N / NI~~
~~**If your aim for this study is to assess the effect of starting and adhering to intervention, answer questions 4.3 to 4.6**~~		
~~4.3. Were important co-interventions balanced across intervention groups?~~		~~Y / PY / PN / N / NI~~
~~4.4. Was the intervention implemented successfully for most participants?~~		~~Y / PY / PN / N / NI~~
~~4.5. Did study participants adhere to the assigned intervention regimen?~~		~~Y / PY / PN / N / NI~~
~~4.6. **If N/PN to 4.3, 4.4 or 4.5**: Was an appropriate analysis used to estimate the effect of starting and adhering to the intervention?~~		~~NA / Y / PY / PN / N / NI~~
~~**Risk of bias judgment**~~		
~~Optional: What is the predicted direction of bias due to deviations from the intended interventions?~~		

Bias due to missing data : NOTE: Here, missing participant data is evaluated starting at recruitment and excluding the rate of participation in recruitment that has been taken into account in the selection bias analysis.		
5.1 Were outcome data available for all, or nearly all, participants?	**YES**: If participation at follow-up is 95% and over and /or data is complete for 95% of participants. Data complete for 90% of participants with a comparison between those included and excluded showing similarity for age, sex and SES or for exposure and for the outcome will be considered adequate.<br>**NO:** If less than 90% of participants are included in the analysis or less than 95% without comparison or with a comparison showing differences	Y / PY / PN / N / NI
5.2 Were participants excluded due to missing data on intervention status?	**YES**: If there were missing data on exposure<br>**NO:** If there are no missing data on the exposure	Y / PY / PN / N / NI
5.3 Were participants excluded due to missing data on other variables needed for the analysis?	**YES:** If there were missing data on covariates<br>**NO:** If there are no missing data on covariates	Y / PY / PN / N / NI
5.4 **If PN/N to 5.1, or Y/PY to 5.2 or 5.3**: Are the proportion of participants and reasons for missing data similar across interventions?	**YES:** If there is a comparison between included and excluded participants due to missing data that shows participants are similar for all three important confounders or for exposure and outcome<br>**NO:** If the comparison shows that the included and excluded are different<br>**NI:** if no information is provided on the differences between the included and the excluded	NA / Y / PY / PN / N / NI
5.5 **If PN/N to 5.1, or Y/PY to 5.2 or 5.3**: Is there evidence that results were robust to the presence of missing data?	**YES:** if a sensitivity analysis was performed to account for missing data (multiple imputation, inverse probability weighting) and the results are similar to the main analysis, or the results are different but the interpretation is done on the sensitivity analysis and not on the main analysis.<br>**NO:** if no sensitivity analysis is done for missing data	NA / Y / PY / PN / N / NI

**Risk of bias judgment**	**Low:** (i) Data were **reasonably complete**; (95% or 90% with demonstrations that they are similar or an analysis was done for missing data) *or*<br>(ii) Proportions of and reasons for missing participants were **similar across intervention groups**;<br>*or*<br>(iii) The **analysis addressed missing data and is likely to have removed any risk of bias**.<br>**Moderate (between 94 (or 89) and 80% at follow-up, can go down to 75% if a comparison shows that they are similar):**<br>(i) Proportions of and reasons for missing participants differ slightly across intervention groups;<br>*and*<br>(ii) The analysis is unlikely to have removed the risk of bias arising from the missing data.<br>**Serious (between 79% (or 74%) and 50% at follow-up with comparison):**<br>(i) Proportions of missing participants differ substantially across interventions; *or* Reasons for missingness differ substantially across interventions;<br>*and*<br>(ii) The analysis is unlikely to have removed the risk of bias arising from the missing data; *or* Missing data were addressed inappropriately in the analysis; *or* The nature of the missing data means that the risk of bias cannot be removed through appropriate analysis.<br>**Critical (<50%):**<br>(i) (Unusual) There were critical differences between interventions in participants with missing data;<br>*and*<br>(ii) Missing data were not, or could not, be addressed through appropriate analysis	Low / Moderate / Serious / Critical / NI
~~Optional: What is the predicted direction of bias due to missing data?~~		~~Favours experimental / Favours comparator / Towards null /Away from null / Unpredictable~~

Bias in measurement of outcomes		
6.1 Could the outcome measure have been influenced by knowledge of the intervention received?	**NO:** Obtained objectively by clinical evaluation or register<br>**PN: S**ome of the diagnoses are obtained by self-reported questionnaire.<br>**YES:** Diabetes diagnoses obtained only by self-reported questionnaire	Y / PY / PN / N / NI
6.2 Were outcome assessors aware of the intervention received by study participants?	**NO:** If the people responsible for collecting data in the register do not know the status of the exposure.<br>**YES**: if the persons responsible for collecting data in the registers know the status of the exposure.	Y / PY / PN / N / NI
6.3 Were the methods of outcome assessment comparable across intervention groups?	**Always YES,** unless the method is different between exposed and unexposed, which would be highly unlikely	Y / PY / PN / N / NI
6.4 Were any systematic errors in measurement of the outcome related to intervention received?	**NO:** Obtained objectively by clinical evaluation or register<br>**PN: S**ome of the diagnoses are obtained by self-reported questionnaire.<br>**YES:** Diabetes diagnoses obtained only by self-reported questionnaire	Y / PY / PN / N / NI

**Risk of bias judgment**	**Low:** (i) The methods of outcome assessment were comparable across intervention groups;<br>*and*<br>(ii) The outcome measure was unlikely to be influenced by knowledge of the intervention received by study participants (i.e. is objective) or the outcome assessors were unaware of the intervention received by study participants;<br>*and*<br>(iii) Any error in measuring the outcome is unrelated to intervention status.<br>**Moderate:** (i) The methods of outcome assessment were comparable across intervention groups;<br>*and*<br>(ii) The outcome measure is only minimally influenced by knowledge of the intervention received by study participants**;**<br>*and*<br>(iii) Any error in measuring the outcome is only minimally related to intervention status.<br>**Serious:**<br>(i) The methods of outcome assessment were not comparable across intervention groups;<br>*or*<br>(ii) The outcome measure was subjective (i.e. vulnerable to influence by knowledge of the intervention received by study participants);<br>*or*<br>(iii) Error in measuring the outcome was related to intervention status.<br>**Critical:**<br>Outcome was assessed by clinical test with a cut-off that is not accepted by the ADA for diabetes definition.	Low / Moderate / Serious / Critical / NI
~~Optional: What is the predicted direction of bias due to measurement of outcomes?~~		Favours experimental / Favours comparator / Towards null /Away from null / Unpredictable

**Bias in selection of the reported result:** This criterion is difficult to apply in our case, because no (or very few) studies have a published protocol and we never have access to the analysis plan. Would still be at moderate risk, therefore will not be evaluated systematically in included studies		
~~Is the reported effect estimate likely to be selected, on the basis of the results, from...~~<br>~~7.1. ... multiple outcome *measurements* within the outcome domain?~~		~~Y / PY / PN / N / NI~~
~~7.2 ... multiple *analyses* of the intervention-outcome relationship?~~		~~Y / PY / PN / N / NI~~
~~7.3 ... different *subgroups*?~~		~~Y / PY / PN / N / NI~~
~~**Risk of bias judgment**~~		~~Low / Moderate / Serious / Critical / NI~~
~~Optional: What is the predicted direction of bias due to selection of the reported result?~~		~~Favours experimental / Favours comparator / Towards null /Away from null / Unpredictable~~

**Overall -**		
**Bias due to confounding**		~~Low~~ / Moderate / Serious / Critical / NI
**Bias in selection of participants into the study**		~~Low~~ / Moderate / Serious / Critical / NI
**Bias in classification of interventions**		Low / Moderate / Serious / Critical / NI
**Bias due to missing data**		Low / Moderate / Serious / Critical / NI
**Bias in measurement of outcomes**		Low / Moderate / Serious / Critical / NI
**Overall**		

This work is licensed under a Creative Commons Attribution-NonCommercial-NoDerivatives 4.0 International License.

Supplementary Text S2. Summary of the quality evaluation of prospective studies on psychosocial work stressors at work and type 2 diabetes, according to Risk of Bias In Non-randomized Studies-Intervention tool (ROBINS-I) criteria.

**Eriksson 2013**		
**Type of bias**	**Classification**	**Reason - explanation**
Bias due to confounding	Serious	Adjusted for age, sex, education level and a post-intervention variable that could have been affected by the intervention (psychological distress)
Bias in selection of participants into the study	Serious	Participation at baseline was 72% without comparison between participants and non-participants
Bias in classification of interventions	Moderate	Slightly shorter questionnaire for decision latitude. No reference provided for the validation of this short version.
Bias due to missing data	Moderate	Complete data for 80% of baseline participants were included in the analyses. Authors provide comparison between included and missing participants showing that those lost to follow-up are relatively similar in terms of exposure, SES, age and sex. No imputation was done.
Bias in measurement of outcomes	Low	Obtained objectively by clinical evaluation
**Overall**	**Serious**	

Garbarino 2018		
**Type of bias**	**Classification**	**Reason - explanation**
Bias due to confounding	Serious	Restricted by sex (men) and adjusted for age, education level and other factors. Education was coded as a binary variable.
Bias in selection of participants into the study	Moderate	Participation at baseline was 99%.
Bias in classification of interventions	Serious	Italian versions of ERI and DC questionnaires are reliable and valid, but the combination of several measurements over time and of the two models has not been validated. No information about exclusion of prevalent cases and no adjustment for high glucose at recruitment.
Bias due to missing data	Moderate	Complete data for 80% of baseline participants were included in the analyses. Authors provide comparison between included and missing participants showing that those lost to follow-up are relatively similar in terms of exposure, personal and socio-economic characteristics. No imputation was done.
Bias in measurement of outcomes	Critical	High fasting glucose was defined by a plasma glucose level of >100 mg/dL (5.6 mmol/L).
**Overall**	Critical	

**Gilbert-Ouimet 2021**		
**Type of bias**	**Classification**	**Reason - explanation**
Bias due to confounding	Serious	Adjusted for age, sex, education level and post-intervention variables that could have been affected by the intervention (chronic medical conditions).
Bias in selection of participants into the study	Serious	Participation at baseline was 63% and total non-response was handled by adjusting the weight of households that responded to the survey to compensate for those who did not respond.
Bias in classification of interventions	Serious	Short version of the job demands scale; partial validation with low Cronbach's α.
Bias due to missing data	Low	Complete data for 95% of baseline participants were included in the analyses.
Bias in measurement of outcomes	Low	Obtained objectively by register (administrative data and physician diagnoses).
**Overall**	**Serious**	

**Heracklides 2009**		
**Type of bias**	**Classification**	**Reason - explanation**
Bias due to confounding	Serious	Adjusted for age and sex, no adjustment for socioeconomic factors.
Bias in selection of participants into the study	Serious	Participation at baseline was 73% without comparison between participants and non-participants
Bias in classification of exposure	Moderate	Intervention status is well defined. Shorter version of demand scale was validated with good α.
Bias due to missing data	Moderate	Complete data for 82% of baseline participants were included in the analyses. Authors provide comparison between included and missing participants showing that those lost to follow-up are rather different in terms of exposure, age and/or sex. No imputation was done.
Bias in measurement of outcomes	Moderate	The methods of outcome assessment were comparable across intervention groups, but some of the cases were ascertained objectively by clinical evaluation, while some were ascertained by self-reported questionnaire.
**Overall**	Serious	

Heraclides 2012		
**Type of bias**	**Classification**	**Reason - explanation**
Bias due to confounding	Serious	Stratified by sex, adjusted for age, employment grade and a post-intervention variable that could have been affected by the intervention (diet pattern).
Bias in selection of participants into the study	Serious	Participation at baseline was 73% without comparison between participants and non-participants
Bias in classification of interventions	Moderate	Intervention status is well defined. Shorter version of demand scale was validated with good α.
Bias due to missing data	Serious	Complete data for 72% of baseline participants were included in the analyses. Authors provide comparison between included and missing participants showing that those lost to follow-up are rather different in terms of exposure, SES, age and sex. No treatment for missing data was done.
Bias in measurement of outcomes	Moderate	The methods of outcome assessment were comparable across intervention groups, but some of the cases were ascertained objectively by clinical evaluation, while some were ascertained by self-reported questionnaire.
**Overall**	Serious	

Hino 2016		
**Type of bias**	**Classification**	**Reason - explanation**
Bias due to confounding	Moderate	Restricted by sex (men). Adjusted for age, marital status, job department, employment position and occupation.
Bias in selection of participants into the study	Critical	Participation at baseline was 21% without comparison between participants and non-participants
Bias in classification of interventions	Moderate	Questionnaire validated in Japanese workers for internal consistency.
Bias due to missing data	Critical	Proportions of missing participants differ substantially across interventions: 43% of the baseline participants included in the analysis, without comparison between included and missing participants.
Bias in measurement of outcomes	Critical	Definition very wide, including diabetes defined by HOMA-IR, which is not a method recommended by the ADA.
**Overall**	**Critical**	

**Huth 2014**		
**Type of bias**	**Classification**	**Reason - explanation**
Bias due to confounding	Moderate	Adjusted for age, sex, physical intensity at work: low, moderate, high. Education was coded as binary variable.
Bias in selection of participants into the study	Serious	Participation at baseline was 75% without comparison between participants and non-participants.
Bias in classification of interventions	Low	Validated version of questionnaire.
Bias due to missing data	Serious	Complete data for 73% of baseline participants were included in the analyses, without comparison between included and missing participants.
Bias in measurement of outcomes	Moderate	The methods of outcome assessment were comparable across intervention groups. Self-reported T2DM and the date of diagnosis were validated by hospital records or by contacting the participants’ treating physicians.
**Overall**	**Serious**	

**Kawakami 1999**		
**Type of bias**	**Classification**	**Reason - explanation**
Bias due to confounding	Serious	Restricted by sex (men), adjustment for age, education level, occupation, use of technology, leisure time and physical activity, family history of diabetes and a post-intervention variable that could have been affected by the intervention (BMI).
Bias in selection of participants into the study	Moderate	Participation at baseline was 92% without comparison between participants and non-participants.
Bias in classification of interventions	Serious	Very short questionnaire with one item for each dimension, not validated.
Bias due to missing data	Serious	Complete data for 77% of baseline participants were included in the analyses without comparison between included and missing participants.
Bias in measurement of outcomes	Moderate	Obtained objectively by clinical evaluation, low risk of false positive outcomes. Some risk of false negative outcomes due to triage by urine insulin, but this risk is lower because the same test had been conducted annually for 12 years before baseline (exclusion of prevalent cases) and each year during follow-up (incident cases).
**Overall**	**Serious**	

**Kroenke 2007**		
**Type of bias**	**Classification**	**Reason - explanation**
Bias due to confounding	Moderate	Restricted by sex (women) and profession (nurses), adjusted for age.
Bias in selection of participants into the study	Serious	Participation at baseline was 75% without comparison between participants and non-participants.
Bias in classification of interventions	Low	Job strain was measured by the well-validated 27-item Karasek Job Content Questionnaire.
Bias due to missing data	Serious	Complete data for 73% of baseline participants were included in the analyses, with a comparison between included and missing participants that shows they are similar for all three important confounders, for exposure and for outcome.
Bias in measurement of outcomes	Moderate	Self-reported diabetes with validation (98%) in a sub-sample.
**Overall**	**Serious**	

Kumari 2004		
**Type of bias**	**Classification**	**Reason - explanation**
Bias due to confounding	Serious	Adjusted for age, length of follow-up, employment grade, ethnic group and a post-intervention variable that could have been affected by the intervention (ECG abnormalities).
Bias in selection of participants into the study	Serious	Participation at baseline was 73% without comparison between participants and non-participants.
Bias in classification of interventions	DC (Moderate); ERI: (Serious)	DC model: Intervention status is well defined**;** a slightly shorter version of the demand scale was validated with good α.<br>ERI model: Unknown number of items. According to Bosma et al 1998: "As there was no original measurement of effort-reward imbalance at phase 1, proxy measures (available from the authors) had to be constructed for the crucial components of the model."
Bias due to missing data	Moderate	Complete data for 82% of baseline participants were included in the analyses, without a comparison between included and missing participants.
Bias in measurement of outcomes	Moderate	The methods of outcome assessment were comparable across intervention groups, but some of the cases were ascertained objectively by clinical evaluation, while some were ascertained by self-reported questionnaire.
**Overall**	**Serious**	

**Mortensen-Gazel 2017**		
**Type of bias**	**Classification**	**Reason - explanation**
Bias due to confounding	Moderate	Adjusted by age, gender, marital status, occupational grade and follow-up duration
Bias in selection of participants into the study	Critical	According to Mortensen, response rate in 2000 was 71% (compared to originally recruited cohort), but according to https://www.hal.inserm.fr/inserm-00488925, PR was 45% at the original recruitment in 1989. No information about analyses comparing participants and non-participants.
Bias in classification of interventions	DC (Low), Social support (Serious)	DC: Shorter version of questionnaire with reference for validation.<br>Social support: only two questions without validation.
Bias due to missing data	Critical	Complete data for 51% of baseline participants were included in the analyses, without a comparison between included and missing participants.
Bias in measurement of outcomes	Serious	The methods of outcome assessment were comparable across intervention groups, but ascertained by self-reported questionnaire.
**Overall**	**Critical**	

Mortensen-SLOSH 2017		
**Type of bias**	**Classification**	**Reason - explanation**
Bias due to confounding	Moderate	Adjusted by age, gender, marital status, occupational grade and follow-up duration
Bias in selection of participants into the study	Critical	According to Magnusson Hanson et al., 2018, Int J Epidemiol, 691–692i: "The numbers of participants in SWES 2003-11 have varied over the years: 9214 in 2003, 9703 in 2005, 7729 in 2007, 6354 in 2009 and 7926 in 2011, representing about 50-64% of the individuals invited to LFS."<br>According to Mortensen et al., 11 441 of 18 914 SWES participants responded at SLOSH baseline: 11 441 / 18914 = 61%.<br>Taking these two numbers together, participation at baseline was 39% or less (≤ 64% * 61%) without comparison between participants and non-participants.
Bias in classification of interventions	DC (Low), Social support (Serious)	Prevailing cases of T2DM were excluded.<br>DC: Intervention status well defined.<br>Social support: short questionnaire with two items without validation.
Bias due to missing data	Critical	Complete data for 46% of baseline participants were included in the analyses, without a comparison between included and missing participants.
Bias in measurement of outcomes	Moderate	Diagnoses were obtained by self-reported questionnaire and supplemented with information on diabetes from hospital admissions.
**Overall**	**Critical**	

**Mortensen-Whitehall 2017**		
**Type of bias**	**Classification**	**Reason - explanation**
Bias due to confounding	Moderate	Adjusted by age, gender, marital status, occupational grade and follow-up duration.
Bias in selection of participants into the study	Serious	Participation at baseline was 73% without comparison between participants and non-participants.
Bias in classification of interventions	DC (Moderate), Social support (Serious)	DC: Intervention status was well-defined. A shorter version of the demand scale was validated with good κ (according to Fransson et al. (2012), κ = 0.83-0.93) and α (according to Heraclides et al., 2009, Cronbach's α 0.67).<br>Social support: short questionnaire with two items, no validation.
Bias due to missing data	Serious	Complete data for 77% of baseline participants were included in the analyses, without a comparison between included and missing participants.
Bias in measurement of outcomes	Moderate	The methods of outcome assessment were comparable across intervention groups, but some of the cases were ascertained objectively by clinical evaluation, while some were ascertained by self-reported questionnaire.
**Overall**	**Serious**	

Mutambudzi 2016		
**Type of bias**	**Classification**	**Reason - explanation**
Bias due to confounding	Critical	Adjusted by education level, race, gender, occupational category, marital status, insurance coverage. No adjustment for age. Adjusted for post-intervention variables that could have been affected by the intervention (BMI, physical activity, alcohol use, hypertension, working hours).
Bias in selection of participants into the study	Serious	Participation at baseline was 74% with a comparison between participants and non-participants. Selection into the study may have been related to intervention and outcome.
Bias in classification of interventions	Serious	Shorter version without information on the validity of the modified JCQ questionnaire, which was a combination of Karasek and Quinn models.
Bias due to missing data	Critical	Complete data of 19% or 50% of baseline participants were included in the analyses, reasons for exclusion unclear. Only 56 participants with missing data were analyzed: “Participants with missing data on the independent variables were excluded from the final multivariate survival analyses. (n = 56, 3.9%). These participants were more likely to be working in high strain jobs at baseline, older, and women.”
Bias in measurement of outcomes	Serious	All of the diagnoses were obtained by self-reported questionnaire.
**Overall**	**Critical**	

Mutambudzi 2018		
**Type of bias**	**Classification**	**Reason - explanation**
Bias due to confounding	Moderate	Adjusted by age, sex, race/ethnicity, education level, and marital status.
Bias in selection of participants into the study	Serious	Participation at baseline was 74% without comparison between participants and non-participants. Selection into the study may have been related to intervention and outcome.
Bias in classification of interventions	Serious	Short version of ERI without information on validity.
Bias due to missing data	Critical	Complete data of between 24%-59% of baseline participants were included in the analyses, reasons for exclusion is unclear; no comparison between included and missing participants.
Bias in measurement of outcomes	Serious	All the diagnoses were obtained by self-reported questionnaire.
**Overall**	**Critical**	

**Norberg 2007**		
**Type of bias**	**Classification**	**Reason - explanation**
Bias due to confounding	Serious	Matched by age, sex and study year, no matching on socioeconomic status, no adjustment for any covariable.
Bias in selection of participants into the study	Critical	Participation at baseline was 52%. According to Weinehall et al. 1998 (Scandinavian Journal of Primary Health Care, 16:3, 171-176), the lowest age group and lowest income quartile were underrepresented among participants. Cholesterol and diastolic blood pressure were significantly different between participants and non-participants.
Bias in classification of interventions	Low	Intervention status was well-defined. Validated Swedish version of DC model questionnaire.
Bias due to missing data	Moderate	≤5% missing data for outcome and ~6% missing data for exposure, unclear for how many participants *either* exposure *or* outcome were missing.
Bias in measurement of outcomes	Low	Obtained objectively from clinical tests.
**Overall**	**Critical**	

**Nordentoft 2020**		
**Type of bias**	**Classification**	**Reason - explanation**
Bias due to confounding	Moderate	Adjusted for sex, age, cohabitation, young children in the household, SES (highest achieved educational level, divided into three groups (Low, ≤9years; Intermediate, 10–12 years; High, ≥13 years), migration background, survey year and sample method
Bias in selection of participants into the study	Serious	Participation at baseline was 54% with a comparison to show differences between participants and non-participants. According to Johnsen et al, BMJ Open 9 (8) (2019) e027056: *"For armed forces, craft and related trade workers, and skilled agricultural, forestry and fishery workers, the association between job type and participation was strongly attenuated after adjustment for sex and age. Additional adjustment for annual income, education, cohabitation, country of origin and geographical region generally attenuated the association between job type and participation"*
Bias in classification of interventions	Moderate	Intervention status was well defined. A shorter version of the ERI questionnaire was validated with good α.
Bias due to missing data	Low	Complete data for 97% of baseline participants were included in the analyses, without a comparison between included and missing participants.
Bias in measurement of outcomes	Low	Obtained objectively by clinical register (administrative data with Classification of Diseases version 10 (ICD-10) code E11)
**Overall**	**Serious**	

**Nyberg-COPSOQ-I and II**		
**Type of bias**	**Classification**	**Reason - explanation**
Bias due to confounding	Moderate	Adjusted for age, sex, SES (occupational title, register based, categorized as low, intermediate, high or other).
Bias in selection of participants into the study	COPSOQ-I=Serious, COPSOQ-II=Critical	Participation at baseline was 61% and 59% for COPSOQ-I and COPSOQ-II, respectively, without comparison between participants and non-participants.
Bias in classification of interventions	Serious	Short version of job demands (3 items) with substantial agreement with complete version.
Bias due to missing data	COPSOQ-I=Low, COPSOQ-II=Moderate	Complete data for 95% of participants (COPSOQ-I) and 88% (COPSOQ-II), without a comparison between included and missing participants
Bias in measurement of outcomes	COPSOQ-I=Low, COPSOQ-II=Moderate	COPSOQ I: Obtained objectively from registers (hospitalization registers).<br>COPSOQ II: The methods of outcome assessment were comparable across intervention groups, but some of the cases were ascertained objectively from registers, while some were ascertained by self-reported questionnaire.
**Overall**	COPSOQ-I=**Serious**, COPSOQ-II=**Critical**	

Nyberg-DWECS		
**Type of bias**	**Classification**	**Reason - explanation**
Bias due to confounding	Moderate	Adjusted by age, sex, SES socioeconomic status (occupational title, register based, categorized as low, intermediate, high or other).
Bias in selection of participants into the study	Serious	Participation at baseline was 75%, without a comparison between participants and non-participants
Bias in classification of interventions	Serious	Short version (demands 3 items, control 5) with substantial agreement with complete version.
Bias due to missing data	Low	Complete data for 99% of baseline participants were included in the analyses.
Bias in measurement of outcomes	Low	Obtained objectively by register (administrative data mortality and hospitalization registers).
**Overall**	**Serious**	

**Nyberg-FPS**		
**Type of bias**	**Classification**	**Reason - explanation**
Bias due to confounding	Moderate	Adjusted by age, sex, SES socioeconomic status (occupational title, register based, categorized in low intermediate, high or other).
Bias in selection of participants into the study	Serious	Participation at baseline was 68%. According to Kivimäki et al. (2007) Am. J. Publ. Health|97:5 Without a comparison between participants and non-participants
Bias in classification of interventions	Serious	Short version (demands 3 items) with substantial agreement with complete version
Bias due to missing data	Low	Complete data for 95% of baseline participants were included in the analyses
Bias in measurement of outcomes	Moderate	The methods of outcome assessment were comparable across intervention groups, but some of the cases were ascertained objectively from registers, while some were ascertained by self-reported questionnaire.
**Overall**	**Serious**	

**Nyberg-Gazel**		
**Type of bias**	**Classification**	**Reason - explanation**
Bias due to confounding	Moderate	Adjusted by age, sex, SES socioeconomic status (occupational title, register based, categorized in low intermediate, high or other).
Bias in selection of participants into the study	Critical	According to https://www.hal.inserm.fr/inserm-00488925, PR was 45% at the original recruitment in 1989. No information about analyzes comparing participants and non-participants.
Bias in classification of interventions	Low	DC: Shorter version of questionnaire with reference for validation.
Bias due to missing data	Serious	Complete data for 53% of baseline participants were included in the analyses without a comparison between included and missing participants
Bias in measurement of outcomes	Serious	The methods of outcome assessment were comparable across intervention groups, but ascertained by self-reported questionnaire.
**Overall**	**Critical**	

Nyberg-HeSSup		
**Type of bias**	**Classification**	**Reason - explanation**
Bias due to confounding	Moderate	Adjusted by age, sex, SES socioeconomic status (occupational title, register based, categorized as low, intermediate, high or other).
Bias in selection of participants into the study	Critical	Participation at baseline was 40%, without a comparison between participants and non-participants.
Bias in classification of interventions	Low	The original version was complete and validated
Bias due to missing data	Serious	Complete data for 62% of baseline participants were included in the analyses, without a comparison between included and missing participants
Bias in measurement of outcomes	Moderate	The methods of outcome assessment were comparable across intervention groups, but some of the cases were ascertained objectively from administrative registers (hospital and reimbursement), while some were ascertained by self-reported questionnaire.
**Overall**	**Critical**	

**Nyberg-IPAW**		
**Type of bias**	**Classification**	**Reason - explanation**
Bias due to confounding	Moderate	Adjusted by age, sex, SES socioeconomic status (occupational title, register based, categorized as low, intermediate, high or other).
Bias in selection of participants into the study	Serious	Participation at baseline was 76%, without a comparison between participants and non-participants
Bias in classification of interventions	Serious	Short version (demands 2 items) with substantial agreement with complete version
Bias due to missing data	Low	Complete data for 96% of baseline participants were included in the analyses.
Bias in measurement of outcomes	Moderate	The methods of outcome assessment were comparable across intervention groups, but some of the cases were ascertained objectively from administrative registers (hospital), while some were ascertained by self-reported questionnaire.
**Overall**	**Serious**	

Nyberg-PUMA		
**Type of bias**	**Classification**	**Reason - explanation**
Bias due to confounding	Moderate	Adjusted by age, sex, SES socioeconomic status (occupational title, register based), categorized in low intermediate, high or other)
Bias in selection of participants into the study	Moderate	Participation at baseline was 80%, without a comparison between participants and non-participants
Bias in classification of interventions	Serious	Short version (demands 3 items, control 5 items) with substantial agreement with complete version
Bias due to missing data	Low	Complete data for 96% of baseline participants were included in the analyses.
Bias in measurement of outcomes	Low	Obtained objectively from registers (hospitalization)
**Overall**	**Serious**	

Nyberg-SLOSH		
**Type of bias**	**Classification**	**Reason - explanation**
Bias due to confounding	Moderate	Adjusted by age, sex, SES socioeconomic status (occupational title, register based), categorized in low intermediate, high or other)
Bias in selection of participants into the study	Critical	Participation at baseline was 43%, without a comparison between participants and non-participants. According to Magnusson Hanson 2018 Int J Epidemiol, 691–692i: "The numbers of participants in SWES 2003-11 have varied over the years: 9214 in 2003, 9703 in 2005, 7729 in 2007, 6354 in 2009 and 7926 in 2011, representing about 50-64% of the individuals invited to LFS."<br>According to Mortensen, 18 914 2003-2005 SWES participants were eligible for SLOSH 2006-2008.<br>According to Nyberg, 12 646 (5985 in 2006 + 6751 in 2008) of the SWES participants responded at SLOSH baseline.<br>12 646 / 18914 = 67%<br>**Total: <= 64% * 66% = 39%**
Bias in classification of interventions	Low	the original version was complete and validated
Bias due to missing data	Serious	Complete data for at least 63% of baseline participants were included in the analyses (based on Hasson et al., 2009 J Epidemiol Community Health 2010;64:453e460). No comparison between included and missing participants.
Bias in measurement of outcomes	Moderate	The methods of outcome assessment were comparable across intervention groups, but some of the cases were ascertained objectively from administrative registers (hospital), while some were ascertained by self-reported questionnaire.
**Overall**	**Critical**	

Nyberg-Still Working

Type of bias	Classification	Reason - explanation
Bias due to confounding	Moderate	Adjusted by age, sex, SES socioeconomic status (occupational title, register based), categorized in low intermediate, high or other)
Bias in selection of participants into the study	Serious	Participation at baseline was 76%, without a comparison between participants and non-participants
Bias in classification of interventions	Serious	Short version (demands 2 items, control 5 items) with substantial agreement with complete version.
Bias due to missing data	Low	Complete data for 98% of baseline participants were included in the analyses.
Bias in measurement of outcomes	Low	Obtained objectively by register (administrative data reimbursement and hospitalization).
**Overall**	**Serious**	

Nyberg-Whitehall II		
**Type of bias**	**Classification**	**Reason - explanation**
Bias due to confounding	Moderate	Adjusted by age, sex, SES socioeconomic status (occupational title, register based), categorized in low intermediate, high or other)
Bias in selection of participants into the study	Serious	Participation at baseline was 73% without comparison between participants and non-participants.
Bias in classification of interventions	Moderate	Intervention status was well-defined. A shorter version of the demand scale was validated with good κ (according to Fransson et al. (2012), κ = 0.83-0.93) and α (according to Heraclides et al., 2009, Cronbach's α 0.67).
Bias due to missing data	Moderate	Complete data for 81% of baseline participants were included in the analyses, without a comparison between included and missing participants.
Bias in measurement of outcomes	Moderate	The methods of outcome assessment were comparable across intervention groups, but some of the cases were ascertained objectively by clinical evaluation, while some were ascertained by self-reported questionnaire.
**Overall**	**Serious**	

**Nyberg-WOLF N and WOLF S**		
**Type of bias**	**Classification**	**Reason - explanation**
Bias due to confounding	Moderate	Adjusted by age, sex, SES socioeconomic status (occupational title, register based, categorized as low, intermediate, high or other)
Bias in selection of participants into the study	Moderate	Participation at baseline was 82% together according to Alfredsson et al. (2002). Without comparison between participants and non-participants.
Bias in classification of interventions	Low	The original scales of job demand and job control from WOLF N was complete and validated
Bias due to missing data	Low	Complete data for 98% of baseline participants were included in the analyses.
Bias in measurement of outcomes	Moderate	The methods of outcome assessment were comparable across intervention groups, but some of the cases were ascertained objectively from administrative registers (hospital), while some were ascertained by self-reported questionnaire.
**Overall**	**Moderate for both**	

Pan 2017		
**Type of bias**	**Classification**	**Reason - explanation**
Bias due to confounding	Moderate	Adjusted for sex, age, education level, vital status and follow-up
Bias in selection of participants into the study	Serious	Participation at baseline was 73% without comparison between participants and non-participants.
Bias in classification of interventions	Serious	Job strain not measured individually but obtained through a job exposure matrix based on job titles.
Bias due to missing data	Moderate	Complete data for 88% of baseline participants were included in the analyses, without comparison between included and missing participants. Multiple imputation with similar results according to the authors.
Bias in measurement of outcomes	Moderate	Some diagnoses were obtained objectively from clinical evaluation and register (administrative data, medical records in Stockholm) and some of the diagnoses were obtained from self-reported questionnaires.
**Overall**	**Serious**	

Smith 2012		
**Type of bias**	**Classification**	**Reason - explanation**
Bias due to confounding	Serious	Stratified for sex, adjusted for age, education level, marital status, ethnicity, immigration status, urban or rural and also for post-intervention variables that could have been affected by the intervention (chronic diseases, activity limitation at work due to health problems).
Bias in selection of participants into the study	Moderate	According to https://www23.statcan.gc.ca/imdb/p2SV.pl?Function=getSurvey&Id=3359, participation at baseline was 84%. Non-participation was corrected through weighting.
Bias in classification of interventions	Serious	Shorter versions of job demands (2 items), job control (5 items) and social support (3 items) questionnaires were validated with reasonable α.
Bias due to missing data	Moderate	Complete data for 89.6% of baseline participants were included in the analyses, with a comparison between included and missing participants. All analyses were weighted to account for the probability of selection into the original sample and non-response
Bias in measurement of outcomes	Low	Obtained objectively from administrative register: 1 hospitalization or 2 reimbursement requests in 2 years (published validation algorithm).
**Overall**	**Serious**	

Souza Santos 2020		
**Type of bias**	**Classification**	**Reason - explanation**
Bias due to confounding	Serious	Stratified by sex. Adjusted for age, weekly workload and education level (coded as a binary variable) and also for a post-intervention variable that could have been affected by the intervention variable (work shift).
Bias in selection of participants into the study	Critical	Participation at baseline was 29%, according to Schmidt 2019 et al https://www.thelancet.com/journals/landia/article/PIIS2213-8587(19)30058-0/fulltext, without comparison between participants and non-participants.
Bias in classification of interventions	Low	Intervention status was well defined: complete and validated versions of questionnaires for the DCS and ERI models.
Bias due to missing data	Moderate	Complete data for 86% of baseline participants were included in the analyses, without a comparison between included and missing participants.
Bias in measurement of outcomes	Low	Obtained objectively by clinical evaluation.
**Overall**	**Critical**	

Toker 2012		
**Type of bias**	**Classification**	**Reason - explanation**
Bias due to confounding	Serious	Adjusted for age, sex, education, follow-up time, family history of type 2 diabetes and a post-intervention variable that could have been affected by the intervention (BMI).
Bias in selection of participants into the study	Moderate	Participation at baseline was 92% without comparison between participants and non-participants
Bias in classification of interventions	Moderate	Intervention status was well defined: use of validated questionnaires, but without validation of the translation.
Bias due to missing data	Serious	Complete data for 55% of baseline participants were included in the analyses, with information showing that the included and excluded are different.
Bias in measurement of outcomes	Moderate	Some diagnoses were obtained objectively from register (administrative data), and some of the diagnoses were obtained from self-reported questionnaires.
**Overall**	**Serious**	

Yamaguchi 2018		
**Type of bias**	**Classification**	**Reason - explanation**
Bias due to confounding	Serious	Adjusted for age, sex, site, family structure, marital status, occupational category (blue collar or white collar), work status and post-intervention variables that could have been affected by the intervention (components of metabolic syndrome).
Bias in selection of participants into the study	Serious	Participation at baseline was 76% without comparison between participants and non-participants
Bias in classification of interventions	Moderate	Possible reverse causality: prevalent cases only partly excluded (only if two or more criteria for metabolic syndrome were present). Japanese version of questionnaire with confirmed reliability and validity.
Bias due to missing data	Serious	Complete data for 56% of baseline participants were included in the analyses without information on comparison between included and missing participants.
Bias in measurement of outcomes	Critical	Outcome was assessed by a clinical test with a cut-off that is not accepted by the ADA diabetes definition: high fasting blood glucose:100 mg/dl.
**Overall**	**Critical**	

Supplemental Figure Legends

Suppl. Figure S1. Flow chart for the selection of the included studies.

Suppl. Figure S2. Effect of high demands on type 2 diabetes mellitus. This analysis considers demands, whether defined dichotomously or in tertiles (highest versus lowest). It was not possible to transform OR or HR into RR since the original studies did not give estimates for the incidence of diabetes in men and women separately; the original values were therefore used. Since the estimates by Kumari et al. (2004) and Heraclides et al. (2009) are from the same cohort, but based on different baselines, both are included in the meta-analysis. Due to this overlap, the width of the confidence intervals might be underestimated. *SE*: standard error. *CI*: confidence interval at 95%.

Suppl. Figure S3. Effect of low job control on type 2 diabetes mellitus. This analysis considers low job control, whether defined dichotomously or in tertiles (highest versus lowest). It was not possible to transform OR or HR into RR since the original studies did not give estimates for the incidence of diabetes in men and women separately; the original values were therefore used. Since the estimates by Kumari et al. (2004) and Heraclides et al. (2009) are from the same cohort, but based on different baselines, both are included in the meta-analysis. Due to this overlap, the width of the confidence intervals might be underestimated. *SE*: standard error. *CI*: confidence interval at 95%.

Suppl. Figure S4. Effect of low social support at work on type 2 diabetes mellitus. This analysis considers low social support at work, whether defined dichotomously or in tertiles (highest versus lowest). It was not possible to transform OR or HR into RR since the original studies did not give estimates for the incidence of diabetes in men and women separately; the original values were therefore used. Since the estimates by Kumari et al. (2004) and Heraclides et al. (2009) are from the same cohort, but based on different baselines, both are included in the meta-analysis. Due to this overlap, the width of the confidence intervals might be underestimated. *SE*: standard error. *CI*: confidence interval at 95%.

Suppl. Figure S5. Effect of job strain on type 2 diabetes mellitus irrespective of risk of bias. Job strain is included either defined as a dichotomous variable or as a contrast between high strain and low strain quadrants, or as continuous variable, or from the objective job strain matrix of Pan et al. (2017); preference was given to dichotomous job strain where available. Male and female subjects in Norberg et al. (2007) were considered separately. For the Gazel, SLOSH and Whitehall II cohorts, only the estimate in Nyberg et al. (2014), which was based on the longest follow-up time, was retained. *SE*: standard error. *CI*: confidence interval at 95%.

Suppl. Figure S6. Effect of job strain on type 2 diabetes mellitus using pooled estimates as published. This analysis retains the pooled estimate published in Nyberg et al. (2014) instead of the individual cohorts and includes all studies irrespective of bias risk. Male and female subjects in Norberg et al. (2007) were considered separately. For the Gazel, SLOSH and Whitehall II cohorts, only the estimate in Nyberg et al. (2014), which was based on the longest follow-up time, was retained. (A) Both sexes. (B) Men only. (C) Women only. *SE*: standard error. *CI*: confidence interval at 95%.

Suppl. Figure S7. Funnel plot for the effect of job strain on type 2 diabetes mellitus using pooled estimates as published. For each cohort represented in Suppl. Figure S5, the relative risk is plotted against its standard error. *Vertical dashed line:* overall relative risk estimate from Suppl Figure S6.

Suppl. Figure S8. Effect of effort-reward imbalance (ERI) on type 2 diabetes mellitus using original measures of effect. The values used for each study are the hazard ratios resp. odds ratios as published without transformation. *SE*: standard error. *CI*: confidence interval at 95%.

Supplemental Figure S1: PRISMA flowchart.

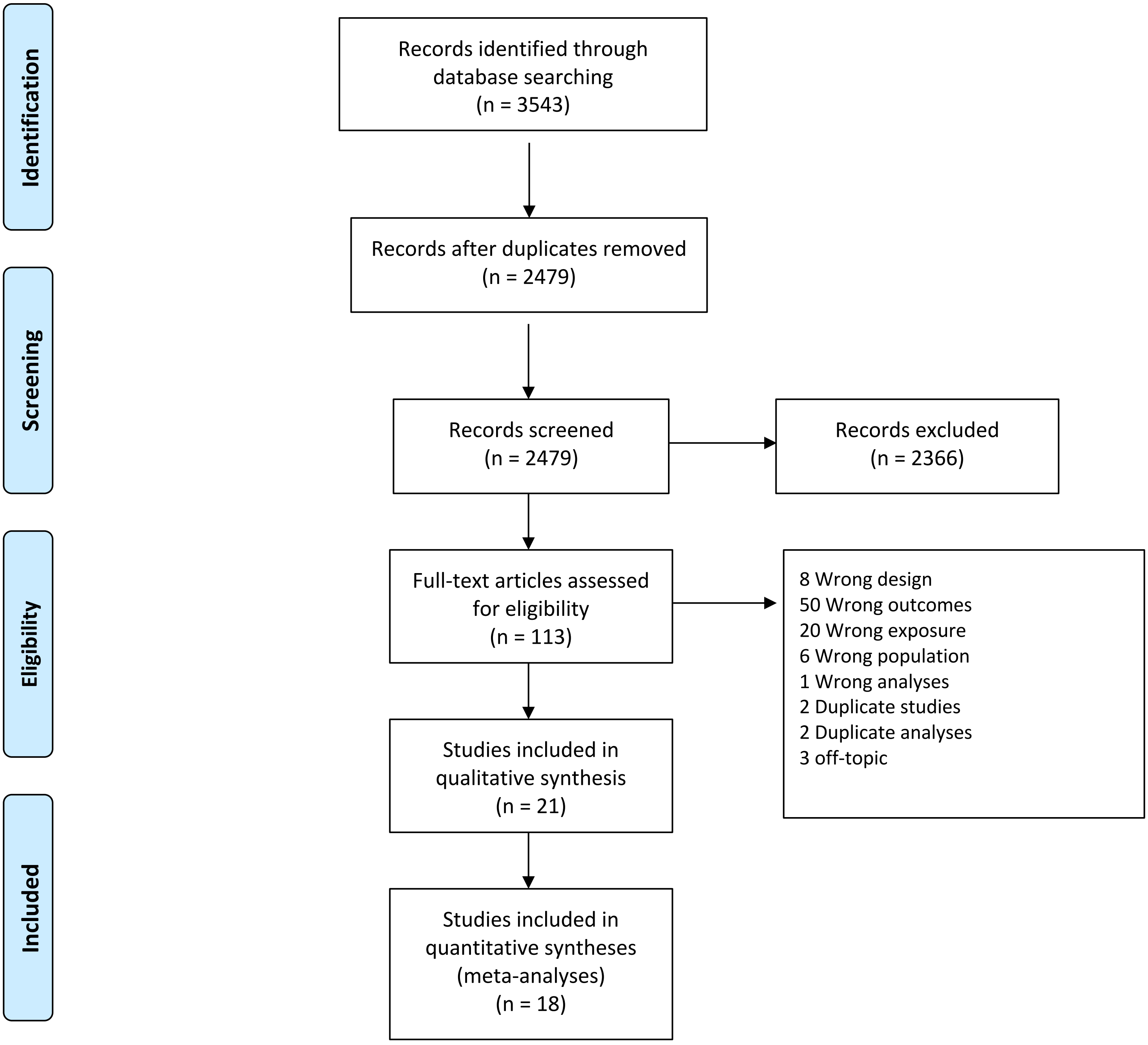

From: Moher D, Liberati A, Tetzlaff J, Altman DG, The PRISMA Group (2009). *P*referred *R*eporting *I*tems for *S*ystematic Reviews and *M*eta-*A*nalyses: The PRISMA Statement. PLoS Med 6(7): e1000097. doi:10.1371/journal.pmed1000097

For more information, visit www.prisma-statement.org.

Study	log (RR)	SE	Weight	Risk Ratio IV, Random, 95% CI
**A. Both sexes**				
Eriksson 2013	−0.36	0.2010	10.9%	0.70 [0.47, 1.04]
Heraclides 2009 (F)	0.06	0.2780	7.3%	1.06 [0.61, 1.83]
Heraclides 2009 (M)	−0.20	0.1090	17.5%	0.82 [0.66, 1.02]
Kumari 2004 (M)	0.10	0.2260	9.5%	1.11 [0.71, 1.73]
Kumari 2004 (F)	−0.53	0.3540	5.1%	0.59 [0.29, 1.18]
Smith 2012 (M)	0.33	0.2180	10.0%	1.39 [0.91, 2.13]
Smith 2012 (F)	0.29	0.2880	7.0%	1.33 [0.76, 2.34]
Souza Santos 2020 (F)	0.88	0.3680	4.8%	2.41 [1.17, 4.96]
Souza Santos 2020 (M)	0.20	0.2520	8.3%	1.22 [0.74, 2.00]
Toker 2012	−0.02	0.0830	19.6%	0.98 [0.83, 1.15]
**Total (95% CI)**			**100.0%**	**1.02 [0.86, 1.22]**

Heterogeneity: $Tau^2 = 0.04$; $Chi^2 = 18.32$, df = 9 (P = 0.03); $I^2 = 51\%$
Test for overall effect: Z = 0.24 (P = 0.81)

Study	log (RR)	SE	Weight	Risk Ratio IV, Random, 95% CI
**B. Men**				
Eriksson 2013	−0.69	0.2800	15.8%	0.50 [0.29, 0.87]
Heraclides 2009	−0.20	0.1090	27.9%	0.82 [0.66, 1.02]
Kumari 2004	0.10	0.2260	19.2%	1.11 [0.71, 1.73]
Smith 2012	0.33	0.2180	19.7%	1.39 [0.91, 2.13]
Souza Santos 2020	0.20	0.2520	17.5%	1.22 [0.74, 2.00]
**Total (95% CI)**			**100.0%**	**0.96 [0.71, 1.30]**

Heterogeneity: $Tau^2 = 0.07$; $Chi^2 = 11.38$, df = 4 (P = 0.02); $I^2 = 65\%$
Test for overall effect: Z = −0.29 (P = 0.77)

Study	log (RR)	SE	Weight	Risk Ratio IV, Random, 95% CI
**C. Women**				
Eriksson 2013	0.18	0.3430	18.9%	1.20 [0.61, 2.35]
Heraclides 2009	0.06	0.2780	23.1%	1.06 [0.61, 1.83]
Kumari 2004	−0.53	0.3540	18.2%	0.59 [0.29, 1.18]
Smith 2012	0.29	0.2880	22.4%	1.33 [0.76, 2.34]
Souza Santos 2020	0.88	0.3680	17.4%	2.41 [1.17, 4.96]
**Total (95% CI)**			**100.0%**	**1.18 [0.79, 1.76]**

Heterogeneity: $Tau^2 = 0.10$; $Chi^2 = 7.92$, df = 4 (P = 0.09); $I^2 = 50\%$
Test for overall effect: Z = 0.83 (P = 0.41)

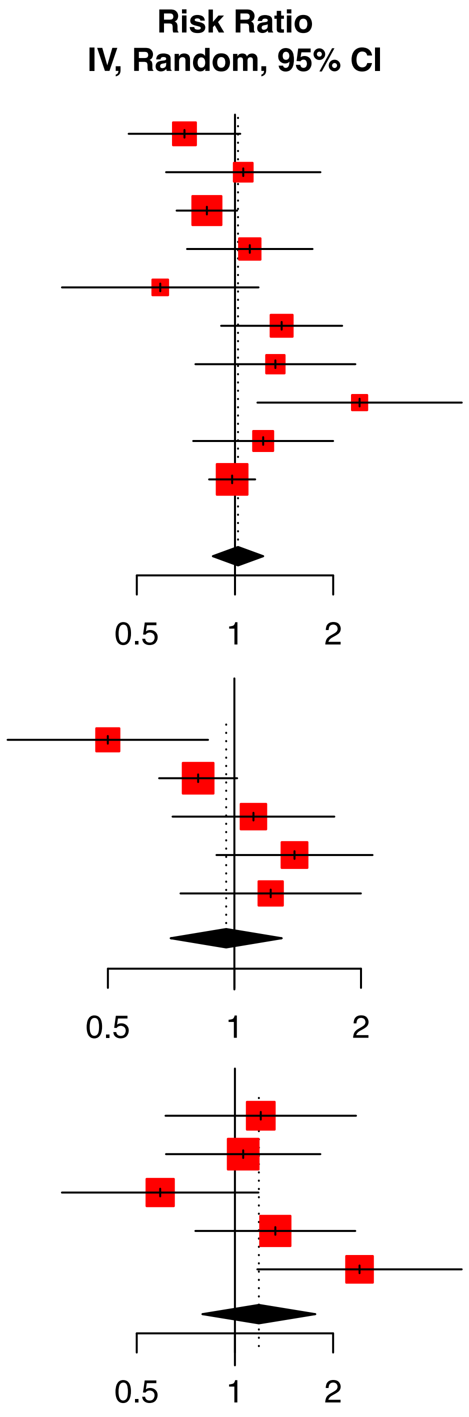

Suppl. Fig. S2. Demand

Study	log (RR)	SE	Weight	Risk Ratio IV, Random, 95% CI
**A. Both sexes**				
Eriksson 2013	0.18	0.2210	9.1%	1.20 [0.78, 1.85]
Heraclides 2009 (F)	0.09	0.1830	11.4%	1.09 [0.76, 1.56]
Heraclides 2009 (M)	−0.15	0.1110	17.3%	0.86 [0.69, 1.07]
Kumari 2004 (M)	−0.26	0.2230	9.0%	0.77 [0.50, 1.19]
Kumari 2004 (F)	−0.20	0.3540	4.6%	0.82 [0.41, 1.64]
Smith 2012 (M)	−0.17	0.2820	6.5%	0.84 [0.48, 1.46]
Smith 2012 (F)	0.77	0.2900	6.3%	2.17 [1.23, 3.83]
Souza Santos 2020 (F)	−0.45	0.2080	9.8%	0.64 [0.43, 0.96]
Souza Santos 2020 (M)	−0.05	0.2280	8.8%	0.95 [0.61, 1.49]
Toker 2012	0.05	0.1120	17.2%	1.05 [0.84, 1.31]
**Total (95% CI)**			**100.0%**	**0.97 [0.82, 1.14]**

Heterogeneity: $Tau^2 = 0.03$; $Chi^2 = 16.23$, df = 9 (P = 0.06); $I^2 = 45\%$
Test for overall effect: Z = −0.42 (P = 0.68)

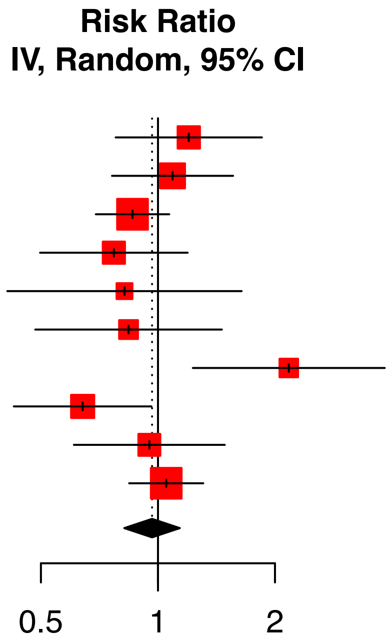

Study	log (RR)	SE	Weight	Risk Ratio IV, Random, 95% CI
**B. Men**				
Eriksson 2013	−0.11	0.2970	7.8%	0.90 [0.50, 1.61]
Heraclides 2009	−0.15	0.1110	56.2%	0.86 [0.69, 1.07]
Kumari 2004	−0.26	0.2230	13.9%	0.77 [0.50, 1.19]
Smith 2012	−0.17	0.2820	8.7%	0.84 [0.48, 1.46]
Souza Santos 2020	−0.05	0.2280	13.3%	0.95 [0.61, 1.49]
**Total (95% CI)**			**100.0%**	**0.86 [0.73, 1.01]**

Heterogeneity: $Tau^2 = 0$; $Chi^2 = 0.47$, df = 4 (P = 0.98); $I^2 = 0\%$
Test for overall effect: Z = −1.82 (P = 0.07)

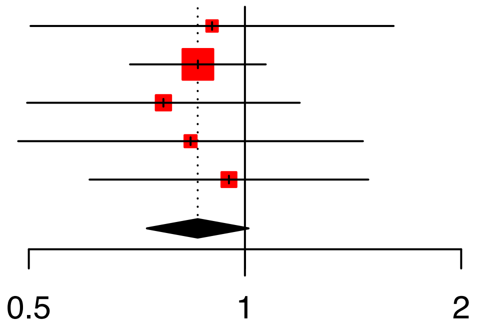

Study	log (RR)	SE	Weight	Risk Ratio IV, Random, 95% CI
**C. Women**				
Eriksson 2013	0.74	0.3780	16.3%	2.10 [1.00, 4.41]
Heraclides 2009	0.09	0.1830	23.9%	1.09 [0.76, 1.56]
Kumari 2004	−0.20	0.3540	17.2%	0.82 [0.41, 1.64]
Smith 2012	0.77	0.2900	19.7%	2.17 [1.23, 3.83]
Souza Santos 2020	−0.45	0.2080	23.0%	0.64 [0.43, 0.96]
**Total (95% CI)**			**100.0%**	**1.17 [0.74, 1.86]**

Heterogeneity: $Tau^2 = 0.20$; $Chi^2 = 15.81$, df = 4 (P < 0.01); $I^2 = 75\%$
Test for overall effect: Z = 0.66 (P = 0.51)

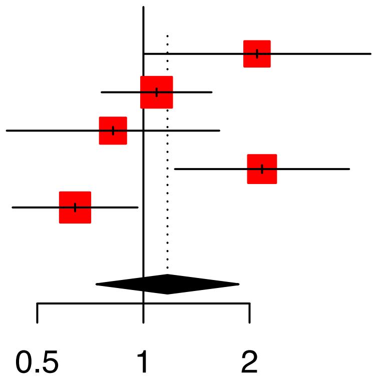

Suppl. Fig. S3. Control

Study	log (RR)	SE	Weight	Risk Ratio IV, Random, 95% CI
**A. Both sexes**				
Heraclides 2009 (F)	0.08	0.1800	8.5%	1.08 [0.76, 1.54]
Heraclides 2009 (M)	0.00	0.1230	11.8%	1.00 [0.79, 1.27]
Kawakami 1999 (M)	0.24	0.4010	2.7%	1.27 [0.58, 2.79]
Kumari 2004 (M)	−0.22	0.2010	7.5%	0.80 [0.54, 1.19]
Kumari 2004 (F)	0.18	0.2550	5.5%	1.20 [0.73, 1.98]
Mortensen−Gazel 2017	0.07	0.1060	12.9%	1.07 [0.87, 1.32]
Mortensen−SLOSH 2017	0.23	0.1460	10.3%	1.26 [0.95, 1.68]
Mortensen−Whitehall 2017	0.24	0.1280	11.5%	1.27 [0.99, 1.63]
Smith 2012 (M)	0.17	0.2880	4.6%	1.19 [0.68, 2.09]
Smith 2012 (F)	−0.84	0.3240	3.9%	0.43 [0.23, 0.81]
Souza Santos 2020 (F)	0.66	0.3480	3.4%	1.93 [0.98, 3.82]
Souza Santos 2020 (M)	0.25	0.2600	5.4%	1.29 [0.77, 2.15]
Toker 2012	−0.24	0.1190	12.0%	0.79 [0.63, 1.00]
**Total (95% CI)**			**100.0%**	**1.05 [0.91, 1.21]**

Heterogeneity: $Tau^2 = 0.03$; $Chi^2 = 23.48$, df = 12 (P = 0.02); $I^2 = 49\%$
Test for overall effect: Z = 0.69 (P = 0.49)

Study	log (RR)	SE	Weight	Risk Ratio IV, Random, 95% CI
**B. Men**				
Heraclides 2009	0.00	0.1230	53.3%	1.00 [0.79, 1.27]
Kawakami 1999	0.24	0.4010	5.0%	1.27 [0.58, 2.79]
Kumari 2004	−0.22	0.2010	20.0%	0.80 [0.54, 1.19]
Smith 2012	0.17	0.2880	9.7%	1.19 [0.68, 2.09]
Souza Santos 2020	0.25	0.2600	11.9%	1.29 [0.77, 2.15]
**Total (95% CI)**			**100.0%**	**1.01 [0.85, 1.21]**

Heterogeneity: $Tau^2 = 0$; $Chi^2 = 2.88$, df = 4 (P = 0.58); $I^2 = 0\%$
Test for overall effect: Z = 0.16 (P = 0.87)

Study	log (RR)	SE	Weight	Risk Ratio IV, Random, 95% CI
**C. Women**				
Heraclides 2009	0.08	0.1800	30.0%	1.08 [0.76, 1.54]
Kumari 2004	0.18	0.2550	26.1%	1.20 [0.73, 1.98]
Smith 2012	−0.84	0.3240	22.5%	0.43 [0.23, 0.81]
Souza Santos 2020	0.66	0.3480	21.3%	1.93 [0.98, 3.82]
**Total (95% CI)**			**100.0%**	**1.02 [0.62, 1.68]**

Heterogeneity: $Tau^2 = 0.18$; $Chi^2 = 10.95$, df = 3 (P = 0.01); $I^2 = 73\%$
Test for overall effect: Z = 0.08 (P = 0.93)

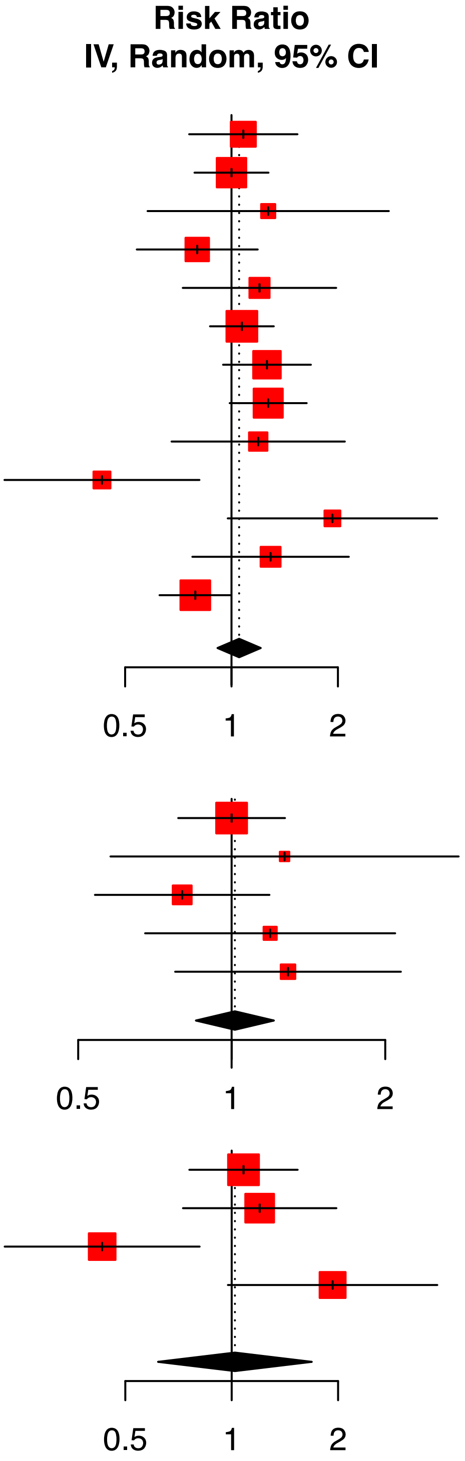

Suppl. Fig. S4. Support

Study	TE	SE	Weight	Risk Ratio IV, Random, 95% CI
Eriksson 2013	−0.22	0.2440	2.1%	0.80 [0.50, 1.29]
Gilbert−Ouimet 2021	0.06	0.1150	9.7%	1.06 [0.85, 1.33]
Huth 2014	0.22	0.1460	6.0%	1.24 [0.93, 1.65]
Kawakami 1999 (M)	0.29	0.5000	0.5%	1.34 [0.50, 3.57]
Kroenke 2007 (F)	0.12	0.1500	5.7%	1.13 [0.84, 1.52]
Mutambudzi 2016	0.55	0.2360	2.3%	1.73 [1.09, 2.75]
Norberg 2007 (M)	0.00	0.3540	1.0%	1.00 [0.50, 2.00]
Norberg 2007 (F)	1.03	0.4930	0.5%	2.80 [1.07, 7.36]
Nyberg−COPSOQ−I 2014	0.05	0.3790	0.9%	1.05 [0.50, 2.21]
Nyberg−COPSOQ−II 2014	0.08	0.6350	0.3%	1.08 [0.31, 3.75]
Nyberg−DWECS 2014	0.43	0.2890	1.5%	1.53 [0.87, 2.70]
Nyberg−FPS 2014	0.16	0.0760	22.1%	1.17 [1.01, 1.36]
Nyberg−Gazel 2014	0.08	0.1050	11.6%	1.08 [0.88, 1.33]
Nyberg−HeSSup 2014	0.06	0.2310	2.4%	1.06 [0.67, 1.67]
Nyberg−IPAW 2014	0.44	0.3130	1.3%	1.55 [0.84, 2.86]
Nyberg−PUMA 2014	0.31	0.5090	0.5%	1.36 [0.50, 3.69]
Nyberg−SLOSH 2014	0.31	0.3580	1.0%	1.37 [0.68, 2.76]
Nyberg−Still Working 2014	0.12	0.0990	13.0%	1.13 [0.93, 1.37]
Nyberg−Whitehall II 2014	0.31	0.4120	0.8%	1.36 [0.61, 3.05]
Nyberg−WOLF N 2014	0.24	0.2870	1.6%	1.27 [0.72, 2.23]
Nyberg−WOLF S 2014	0.09	0.1190	9.0%	1.09 [0.86, 1.38]
Pan 2017	0.47	0.2050	3.0%	1.60 [1.07, 2.39]
Souza Santos 2020 (F)	0.57	0.3010	1.4%	1.77 [0.98, 3.19]
Souza Santos 2020 (M)	0.02	0.2790	1.6%	1.02 [0.59, 1.76]
**Total (95% CI)**			**100.0%**	**1.17 [1.09, 1.25]**

Heterogeneity: $Tau^2 = 0$; $Chi^2 = 17.49$, df = 23 ($P = 0.78$); $I^2 = 0\%$

Test for overall effect: $Z = 4.34$ ($P < 0.01$)

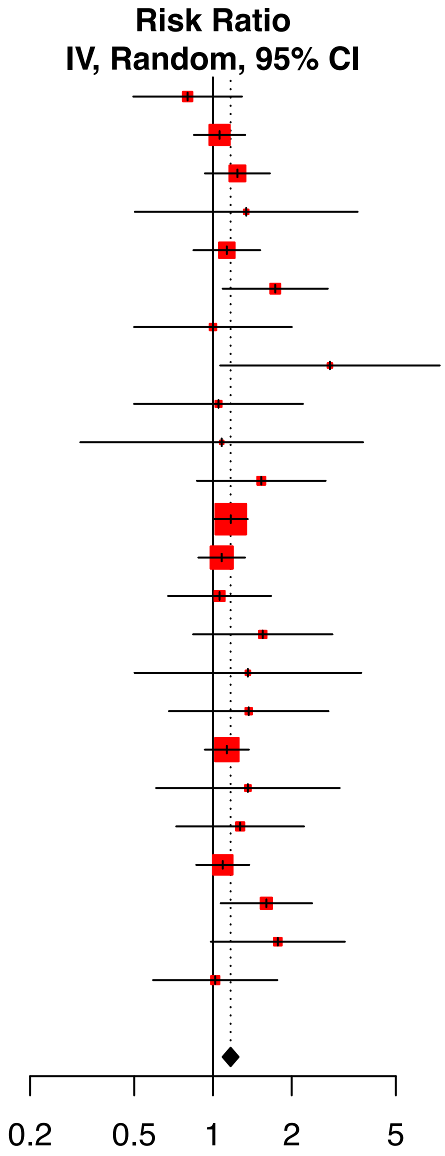

Suppl. Fig. S5. All job strain cohorts

Study	log (RR)	SE	Weight	Risk Ratio IV, Random, 95% CI
**A. Both sexes**				
Eriksson 2013 Q	−0.21	0.2367	4.8%	0.81 [0.51, 1.29]
Gilbert−Ouimet 2021 C	0.06	0.1153	14.9%	1.06 [0.85, 1.33]
Huth 2014 D	0.21	0.1384	11.5%	1.23 [0.94, 1.61]
Kawakami 1999 (M) D	0.29	0.4905	1.2%	1.33 [0.51, 3.48]
Kroenke 2007 (F) Q	0.12	0.1496	10.2%	1.13 [0.84, 1.52]
Mutambudzi 2016 Q	0.54	0.2323	4.9%	1.72 [1.09, 2.71]
Norberg 2007 (M) Q	0.00	0.3524	2.3%	1.00 [0.50, 1.99]
Norberg 2007 (F) Q	1.02	0.4845	1.2%	2.77 [1.07, 7.16]
Nyberg 2014 D	0.14	0.0421	34.7%	1.15 [1.06, 1.25]
Pan 2017 D	0.44	0.1862	7.2%	1.55 [1.08, 2.23]
Souza Santos 2020 (F) D	0.55	0.2888	3.3%	1.74 [0.99, 3.06]
Souza Santos 2020 (M) D	0.02	0.2701	3.8%	1.02 [0.60, 1.73]
**Total (95% CI)**			**100.0%**	**1.19 [1.07, 1.33]**

Heterogeneity: $Tau^2 < 0.01$; $Chi^2 = 14.10$, df = 11 (P = 0.23); $I^2 = 22\%$
Test for overall effect: Z = 3.23 (P < 0.01)

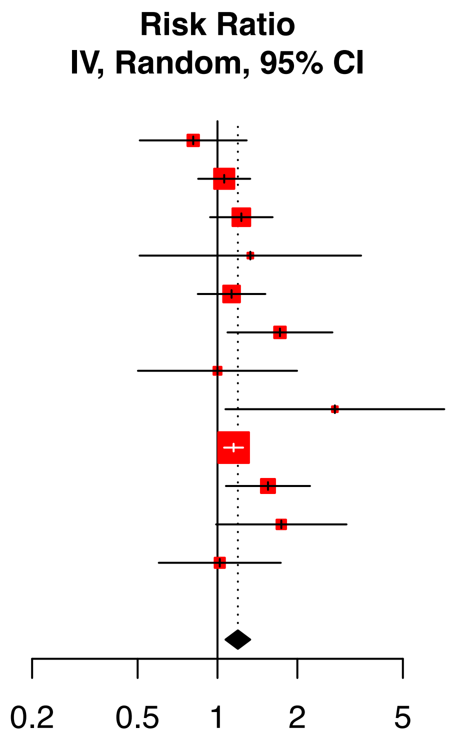

Study	log (RR)	SE	Weight	Risk Ratio IV, Random, 95% CI
**B. Men**				
Eriksson 2013 Q	−0.69	0.2800	13.4%	0.50 [0.29, 0.87]
Gilbert−Ouimet 2021 C	−0.07	0.1560	23.8%	0.93 [0.69, 1.26]
Kawakami 1999 D	0.29	0.5000	5.6%	1.34 [0.50, 3.57]
Norberg 2007 Q	0.00	0.3540	9.7%	1.00 [0.50, 2.00]
Nyberg 2014 D	0.17	0.0600	34.0%	1.19 [1.06, 1.34]
Souza Santos 2020 D	0.02	0.2790	13.5%	1.02 [0.59, 1.76]
**Total (95% CI)**			**100.0%**	**0.97 [0.75, 1.25]**

Heterogeneity: $Tau^2 = 0.05$; $Chi^2 = 11.06$, df = 5 (P = 0.05); $I^2 = 55\%$
Test for overall effect: Z = −0.25 (P = 0.80)

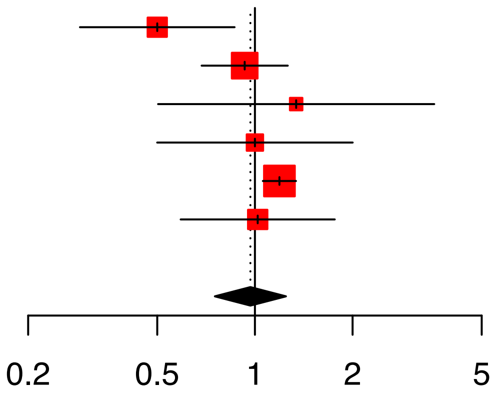

Study	log (RR)	SE	Weight	Risk Ratio IV, Random, 95% CI
**C. Women**				
Eriksson 2013 Q	0.74	0.4270	4.3%	2.10 [0.91, 4.85]
Gilbert−Ouimet 2021 C	0.21	0.1840	17.3%	1.23 [0.86, 1.76]
Kroenke 2007 Q	0.12	0.1500	22.5%	1.13 [0.84, 1.52]
Norberg 2007 Q	1.03	0.4930	3.3%	2.80 [1.07, 7.36]
Nyberg 2014 D	0.12	0.0630	44.6%	1.13 [1.00, 1.28]
Souza Santos 2020 D	0.57	0.3010	8.0%	1.77 [0.98, 3.19]
**Total (95% CI)**			**100.0%**	**1.26 [1.05, 1.51]**

Heterogeneity: $Tau^2 = 0.01$; $Chi^2 = 7.32$, df = 5 (P = 0.20); $I^2 = 32\%$
Test for overall effect: Z = 2.49 (P = 0.01)

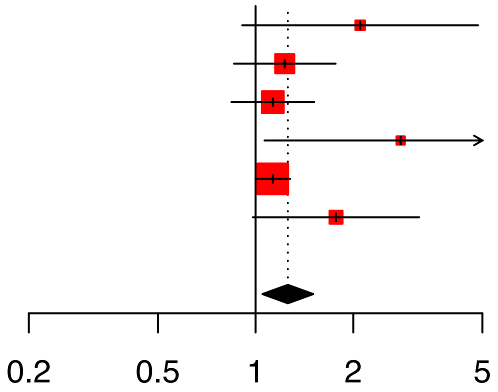

Suppl. Fig. S6. Job strain aggregated cohorts

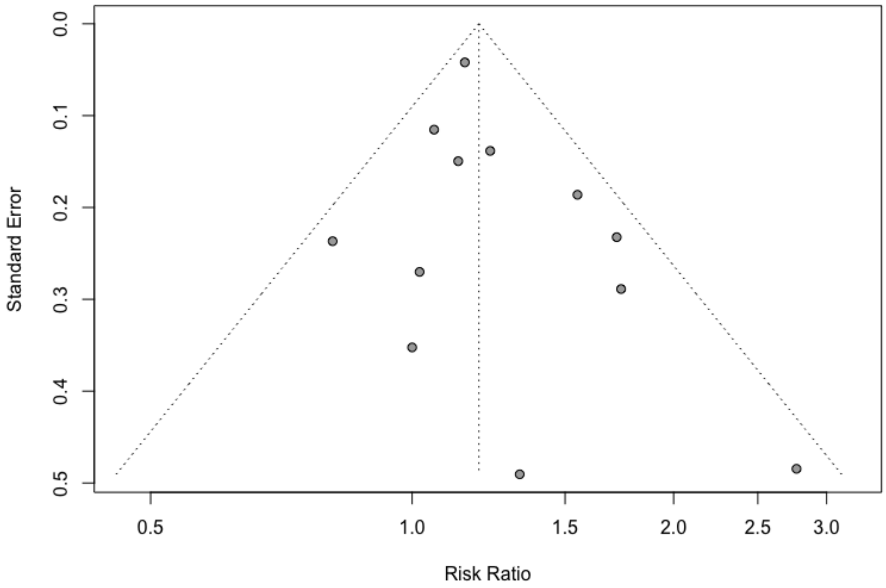

Suppl. Fig. S7. Funnel plot aggregated cohorts

Study	TE	SE	Weight	Risk Ratio IV, Random, 95% CI
Kumari 2004 (M)	0.54	0.2630	7.2%	1.71 [1.02, 2.86]
Kumari 2004 (F)	−0.08	0.3970	3.1%	0.92 [0.42, 2.00]
Mutambudzi 2018	0.17	0.1160	36.9%	1.18 [0.94, 1.48]
Nordentoft 2020	0.24	0.1120	39.6%	1.27 [1.02, 1.58]
Souza Santos 2020 (F)	0.31	0.2650	7.1%	1.36 [0.81, 2.29]
Souza Santos 2020 (M)	0.08	0.2830	6.2%	1.08 [0.62, 1.88]
**Total (95% CI)**			**100.0%**	**1.24 [1.08, 1.43]**

Heterogeneity: $Tau^2 = 0$; $Chi^2 = 2.65$, df = 5 (P = 0.75); $I^2 = 0\%$
Test for overall effect: Z = 3.09 (P < 0.01)

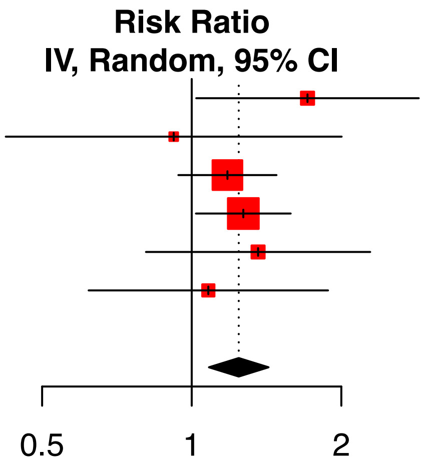

Suppl. Fig. S8. ERI original effect measures
